# Computational modeling reveals key factors driving treatment-free remission in chronic myeloid leukemia patients

**DOI:** 10.1038/s41540-024-00370-4

**Published:** 2024-04-27

**Authors:** Xiulan Lai, Xiaopei Jiao, Haojian Zhang, Jinzhi Lei

**Affiliations:** 1 Institute for Mathematical Sciences, Renmin University of China, Beijing, China; 2 Department of Mathematics, Tsinghua University, Beijing, China; 3 The State Key Laboratory Breeding Base of Basic Science of Stomatology and Key Laboratory of Oral Biomedicine Ministry of Education, School and Hospital of Stomatology, Medical Research Institute, School of Medicine, Wuhan University, Wuhan, China; 4 Frontier Science Center for Immunology and Metabolism, Medical Research Institute, School of Medicine, Wuhan University, Wuhan, China; 5 School of Mathematical Sciences, Center for Applied Mathematics, Tiangong University, Tianjin, China

**Keywords:** Cancer, Applied mathematics

## Abstract

Patients with chronic myeloid leukemia (CML) who receive tyrosine kinase inhibitors (TKIs) have been known to achieve treatment-free remission (TFR) upon discontinuing treatment. However, the underlying mechanisms of this phenomenon remain incompletely understood. This study aims to elucidate the mechanism of TFR in CML patients, focusing on the feedback interaction between leukemia stem cells and the bone marrow microenvironment. We have developed a mathematical model to explore the interplay between leukemia stem cells and the bone marrow microenvironment, allowing for the simulation of CML progression dynamics. Our proposed model reveals a dichotomous response following TKI discontinuation, with two distinct patient groups emerging: one prone to early molecular relapse and the other capable of achieving long-term TFR after treatment cessation. This finding aligns with clinical observations and underscores the essential role of feedback interaction between leukemic cells and the tumor microenvironment in sustaining TFR. Notably, we have shown that the ratio of leukemia cells in peripheral blood (PBLC) and the tumor microenvironment (TME) index can be a valuable predictive tool for identifying patients likely to achieve TFR after discontinuing treatment. This study provides fresh insights into the mechanism of TFR in CML patients and underscores the significance of microenvironmental control in achieving TFR.

## Introduction

During the process of hematopoiesis, hematopoietic stem cells (HSCs) undergo proliferation, expansion, and differentiation, generating diverse mature blood cells across various lineages. These HSCs reside in specialized areas of the bone marrow microenvironment called “hematopoietic niches”^1^. The hematopoietic process is regulated by intrinsic signaling networks and external signals from the bone marrow microenvironment. However, when hematopoietic stem cells or progenitor cells acquire oncogene mutations and clonally expand, it may interrupt normal hematopoiesis and cause malignancies of blood cells.

Chronic myeloid leukemia (CML) is a type of cancer that occurs when a certain type of stem cell in the bone marrow undergoes a genetic change called BCR-ABL1 fusion gene-induced transformation. This change is caused by a chromosomal translocation known as t(9;22), and it transforms the cell into a leukemic stem cell (LSC)^2,3^. The LSC multiplies and produces an excess of immature myeloid lineage cells, mostly granulocytes, in the bone marrow, which causes a clonal myeloproliferative disease. CML progresses slowly and typically goes through three phases while left treatment: chronic phase, accelerated phase, and blast crisis phase. The chronic phase is the initial, relatively inactive stage that may last between 3 and 7 years^4^. CML is usually diagnosed during the chronic phase when there is an abnormally high number of white blood cells in the body. If chronic phase CML is not treated, it will eventually progress to the accelerated phase and/or the blast crisis phase within 5 years^4^. In the accelerated phase, CML cells increase rapidly, and immature blast cells in the blood or the marrow increase (up to 19%). In the blast crisis phase, blast cells may accumulate in other areas of the body, and the number of blasts in the blood or marrow increases to over 20%. Most deaths related to CML occur during the accelerated or blast crisis phase. However, the progression of the disease varies greatly among individuals. Some may remain in the stable chronic phase for up to 20 years, while others may progress within a few months^4^.

CML is a typical disease treated with targeted therapy. Over the past decade, the standard treatment has involved tyrosine kinase inhibitors (TKIs) targeting the BCR-ABL fusion protein. This has significantly improved the life expectancy of patients with CML^5–10^. Initially, TKI therapy aimed to prevent patients from progressing to an accelerated phase or blast crisis, and required lifelong imatinib therapy. Ten-year follow-up studies of imatinib recipients showed a 10-year overall survival rate of around 80%^11,12^. More than 82% of patients treated with imatinib achieved a complete cytogenetic response, defined as no Philadelphia chromosome-positive cell in at least 20 marrow metaphases. With prolonged imatinib therapy, deeper molecular response levels can be achieved. These are measured using real-time quantitative reverse transcriptase polymerase chain reaction (qRT-PCR) to determine the ratio of BCR-ABL1 mRNA to control transcription in peripheral blood (BCR-ABL1/ABL1 ratio). Clinically, a major molecular response corresponds to BCR-ABL1/ABL1 ratio ≤ 0.1%. Deeper molecular responses, such as MR4.0 (BCR-ABL1/ABL1 ratio ≤ 0.01%) and MR4.5 (BCR-ABL1/ABL1 ratio ≤ 0.0032%), and a complete molecular response (BCR-ABL1/ABL1 ratio ≤ 0.001% or undetectable BCR-ABL1 mRNA) are also possible^13,14^. MR4.5 can be achieved in approximately 30−50% of patients treated with imatinib within 5 years and in approximately 40−60% of patients treated with a second-generation TKI^13,14^. Recent studies have shown that a significant proportion of patients who achieve deep molecular responses can achieve treatment-free remission (TFR) after discontinuation of TKI therapy^15,16^.

Several clinical studies have investigated TFR after TKI treatment^17–25^. However, since the concept of TFR is relatively new, little is known about the molecular components that may regulate patient responses^26^. Clinical studies on imatinib treatment^17–21^ have shown that after stopping imatinib in patients with stable undetectable BCR-ABL1 mRNA for at least two years, the overall molecular relapse rate was about 60%, while long-term survival was around 40%. Molecular recurrence mostly occurred within six months after imatinib withdrawal, and late relapses were rarely observed. In the long-term follow-up of the French stop imatinib study^20^, molecular recurrence-free survival was 43% (95% CI, 33% to 52%) at six months and 38% (94% CI, 29% to 47%) at 60 months. Similar results have been observed in studies of second-generation TKIs (nilotinib, dasatinib, and bosutinib)^22–25^, with a 96-week molecular recurrence-free of 49% in the study by Ross et al.^24^ and 53% in the study by Mahon et al.^25^, both of which evaluated the effects of nilotinib discontinuation. In both first- and second-generation TKI studies, most relapsed patients who reinitiated the original TKI regained a major molecular response. Moreover, some patients can achieve a second TFR after a second attempt despite failing the first TKI discontinuation attempt^27^.

Clinical studies showed varying outcomes in patients with CML who discontinued TKI treatment. Some patients relapsed within six months, while others achieved long-term TFR with rare late relapsed cases. Despite TKI treatment, leukemic stem cells can be observed during TFR^24,28^, which suggests that other factors contribute to maintaining long-term TFR. Various parameters may be associated with TFR, such as age, sex^17,29^, Sokal score^17^, duration of TKI treatment^17,18,30,31^, and the immune status^22,23,32–35^. The relationship between leukemia cells and the bone marrow microenvironment is crucial for CML progression^26,36,37^. However, the optimal predictors of successful TFR remain undefined^15,38^.

Mathematical models have been widely used to study the mechanisms that regulate hematopoiesis and leukemia. In particular, in^39–42^, mathematical modeling was employed to investigate the impact of chronic inflammation on the development and progression of chronic Philadelphia-negative myeloproliferative neoplasms, the effect of cytokine dependence of leukemia cells on the course of acute myeloid leukemia, the responses of CML patients to tyrosine kinase inhibitor treatment, and the competitive dynamics of imatinib-resistant CML strains when exposed to second-line medication.

Two separate studies have used mathematical models to investigate the behavior of different types of blood cells in the context of diseases. In Andersen’s work^39,43^, the focus was on stem cells, mature cells, and malignant stem cells with mutations. The study confirmed that chronic inflammation is a significant factor that triggers and drives the progression of chronic Philadelphia-negative myeloproliferative neoplasm disease. In Stiehl’s study^40^, the goal was to understand the impact of cytokine dependence of leukemic cells on acute myeloid leukemia. Results showed that cytokine-independent leukemic cell proliferation could be linked to early relapses and poor overall survival.

The study by Fassoni et al.^41^ discussed a model used to analyze the responses of CML patients to tyrosine kinase inhibitor treatment. The study aimed to explore the influence of different dosing regimens on the treatment outcome. The research revealed that the drug’s efficiency determines the initial response, while the rare activation of leukemic stem cells limits the long-term behavior.

In the study by Woywod et al.^42^, a model was employed to investigate the competitive dynamics among imatinib-resistant CML strains when subjected to second-line medication like dasatinib and nilotinib. The study delved into assessing the impact of mutation rates, initial populations, clonal competition involving differential sensitivities, and the quiescence of stem cells.

There is a scarcity of studies that use mathematical modeling studies to investigate the possibility of TFR for patients with CML who have stopped the treatment. In a recent study, a mathematical model was developed to predict the possibility of relapse during the second attempt at treatment-free remission^44^. The model describes the dynamics of the leukemia cell population in response to TKI treatments and categorizes patients into two groups based on the timing of relapse—early or late. However, the model cannot explain why some patients achieve long-term TFR after treatment discontinuation.

Understanding the clinical outcome of CML requires a good grasp of the dynamics of the leukemic-related hematopoietic system. Mathematical modeling has been used to predict the rapid increase in BCR-ABL1 when treatment is withdrawn, and TFR would not be expected without stem cell-niched interaction^44–47^. In recent years, various mathematical models have been proposed to study how the bone marrow microenvironment or stem cell niche regulates the hematopoiesis dynamics through their influence on the HSC capacity^48–51^. However, these interactions alone cannot explain the outcome of TFR, and other factors must be considered. For example, Fassoni and Glauche developed a mathematical model for CML that incorporates the immune system’s antileukemic effect and applied the model to 21 CML patients who ceased TKI treatment^52,53^. The model provided strong evidence that different immunologic configurations in patients with CML determine their response to therapy cessation.

This study focused on exploring the mechanism underlying TFR in CML patients, considering the interplay between leukemia stem cells and the bone marrow microenvironment. We presented a mathematical model of hematopoiesis that considers both normal and leukemia stem cells (LSCs) and the leukemia-microenvironment crosstalk. We employed a stochastic differential equations model to account for the high heterogeneity of the disease progression among individual patients. We quantified the level of leukemia in patients by measuring the ratio of leukemia cells to normal cells circulating blood. The model incorporates imatinib therapy by increasing the death rate of leukemia cells. By comparing model dynamics with clinical data, we identified model parameters that can reproduce the three stages of CML development. Furthermore, our model simulations allow us to investigate the dynamics of patient responses after imatinib discontinuation, providing evidence of how leukemia-microenvironment crosstalk plays an essential role in predicting TFR for individual patients.

## Results

### Mathematical model of CML evolution dynamics

**Fig. 1:**
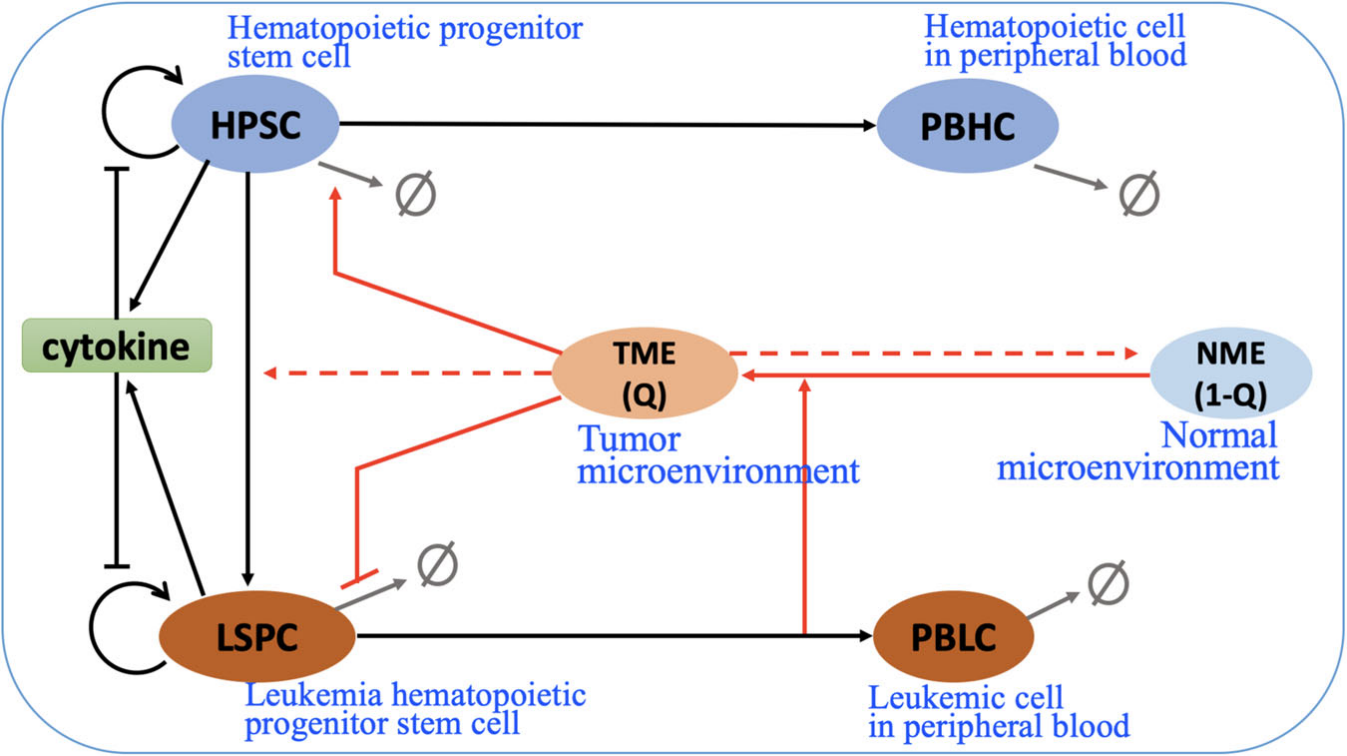
Schematic representation of CML evolution driven by the tumor microenvironment. Black arrows indicate cell interaction/transitions, red arrows show the interactions associated with the microenvironment, and grey arrows represent cell death.

To quantitatively understand the evolution of chronic myeloid leukemia (CML), we established a random differential equation model to study the behavior of hematopoietic stem and progenitor cells (HSPCs) and leukemia stem and progenitor cells (LSPCs) in the bone marrow microenvironment (BMM). In the proposed model, we considered how leukemia cells interact with the surrounding niche, which leads to differences between the normal microenvironment (NME) and the tumor microenvironment (TME). Moreover, we examined the population dynamics of hematopoietic cells in peripheral blood (PBHC) and leukemic cells in peripheral blood (PBLC) to compare with clinical data. A diagram of the interactions is shown in Fig. 1.

In our model illustrated in Fig. 1, we assumed leukemia cells are produced from normal HSPC due to the random occurrence of BCR-ABL fusion genes. Cytokines and intracellular signaling pathways regulate the proliferation of HSPC and LSPC. As the stem cells differentiate into downstream progenitor cells, the progenitor cells proliferate in the bone marrow, and mature cells enter the peripheral blood compartment. Through the model, we quantified the phases of CML through the ratio of leukemic cells present in the peripheral blood.

Leukemia cells interact with their surrounding niche to create a favorable environment for leukemia progression^36,54–56^. Although we did not delve into the specific molecular mechanisms of leukemia-BMM interactions in our model, we introduced a variable *Q*, named the TME index, to represent the microenvironmental condition that can change between NME and TME. We assumed that leukemia cells can cause the transition from a normal microenvironment (NME) to a tumor microenvironment (TME) and that TME promotes the survival of leukemia cells. Additionally, TME represses the survival of HPSC and increases the potential frequency of generating leukemia cells from normal HSPC to LSPC. The red arrows in Fig. 1 illustrate the interactions associated with the microenvironment. For detailed mathematical model formulations, please refer to Methods.

### Model validation with CML evolution

Clinically, CML is classified into three phases based on the number of immature white blood cells (blasts) present in the blood or bone marrow. These phases are chronic phase (CP), accelerated phase (AP), and blast crisis (BC). According to the World Health Organization criteria, these phases are defined as blast percentage of less than 10%, 10% to 20%, and beyond 20%, respectively^9,10,57^. However, the exact disease age from the occurrence of BCR-ABL1 fusion gene is not known for a specific patient^58^. Hence, to validate the proposed model, we suggested a definition for the disease age through the gene expression of a patient. We identified the model parameters and compared the simulated CML progression with the available clinical data, as discussed in Methods.

**Fig. 2:**
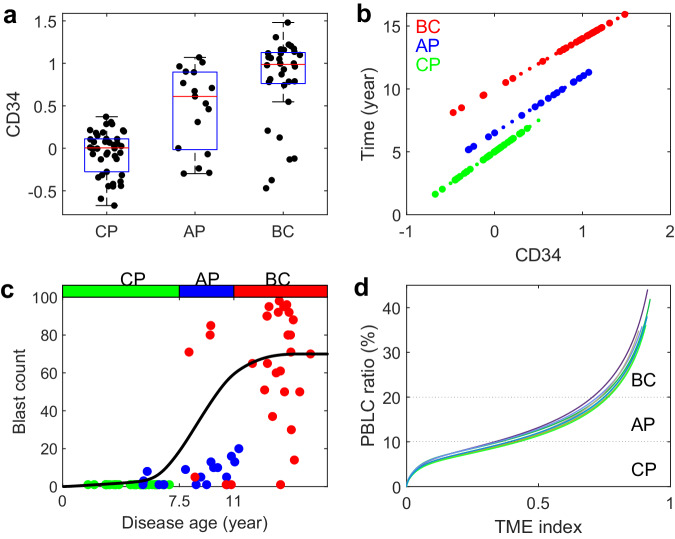
Data analysis of CML evolution. **a** CD34 expression level of patients in three phases of CML evolution: chronic phase (CP), accelerated phase (AP), and blast crisis (BC). **b** Disease age versus CD34 expression level through the equation (1). Red, green, and black dots denote CP, AP, and BC patients, respectively. **c** Evolution of blast counts of patients. Green, blue, and red dots denote CP, AP, and BC patients, respectively. The blue curve represents the average of 1000 sample simulations. **d** PBLC percentage in the peripheral blood versus the TME index during CML evolution without therapy obtained by model simulation.

We analyzed gene expression data in a group of patients with CML to measure the progression of the disease. The study involved 42 patients in the chronic phase, 17 in the accelerated phase, and 32 in the blast crisis phase (32 cases)^59^. Our findings revealed a positive correlation between CD34 expression and the progression of CML (Fig. 2a). To track the progression of the disease, we proposed a disease progression marker through the CD34^+^ expression^58^. We then defined the CML progression age starting from the merge of BCR-ABL1 cells through CD34 gene expression using the following formula (refer to Methods):

1 $${T}_{{{{\rm{disease}}}}\,{{{\rm{age}}}}}=A\times [{{{\rm{CD}}}}34]+B.$$

Here, [CD34] represents the normalized CD34 expression level of a patient. The coefficients (*A*, *B*) were chosen so that the disease ages of patients at CP, AP, and BC phases were distributed separately (refer to Fig. 2b and Supplementary Fig. 2).

The blast count in clinical data indicates the concentration of leukemia cells in the peripheral blood, which is proportional to the number of PBLC in the model. To track the disease progression stages of mixed-population clinical samples, we compared the disease progression of different patients with model simulation by analyzing the blast count versus the CML age (Fig. 2c). Different patients were modeled with parameters sampled over the parameter ranges in Supplementary Table 1. The blue solid curve in Fig. 2c shows the simulated evolution of PBLC ratio after the occurrence of the first LSPC, which was obtained from the average of multiple sample simulations. Both clinical data and simulations reveal three stages of dynamics in the blast counts, characterized by slow development during CP, fast increase during AP, and high-level maintenance during BC.

Figure 2d shows the relationship between the PBLC ratio *R*_PBLC_ in peripheral blood and the TME index during the progression of CML. The ratio *R*_PBLC_ and the TME index changed with the disease progression. During CP, the TME index increased rapidly, while the ratio *R*_PBLC_ increased slowly. During AP, the rate of change in the TME index slowed down, while the ratio *R*_PBLC_ increased more quickly, resulting in the transition from CP to the BC phase. In the BC phase, the rate of increase in the TME index further slowed down, and the ratio *R*_PBLC_ rapidly increased until it reached a saturation level. These findings supported the proposed model and confirmed the three-phase dynamics of CML progression.

### CML progression with imatinib therapy

**Fig. 3:**
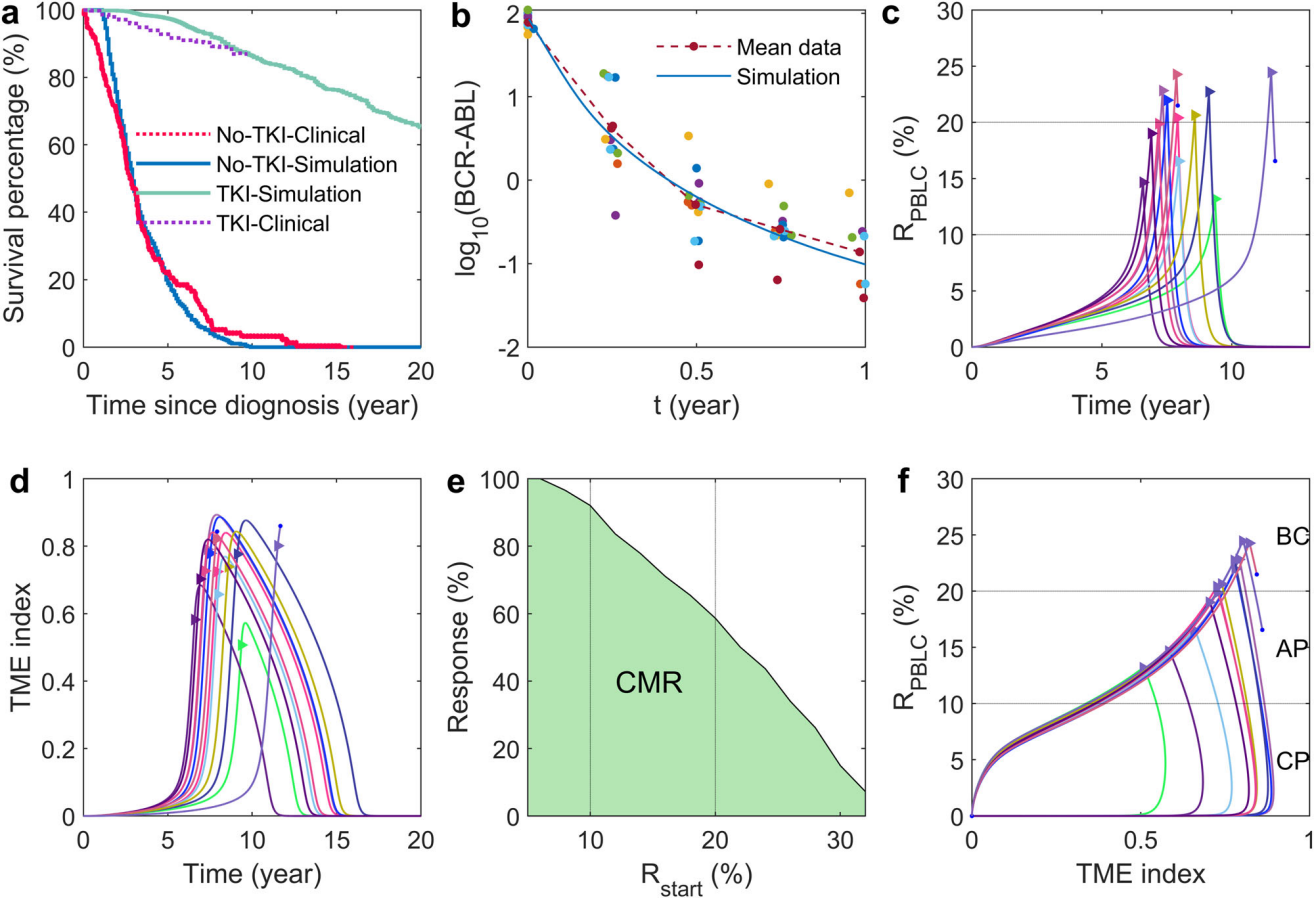
CML evolution for patients under continuous imatinib treatment. **a** Overall survival rates of CML patients. The red dotted line represents data from MD Anderson Cancer Center for patients who received no treatment^60^. The blue line represents the model simulation of patients without treatment. The magenta dotted line represents data from the German CML study group since 1983^62^. The green line represents the simulation of patients who received continuous imatinib treatment. **b** Decreasing of the BCR-ABL1 ratio after imatinib therapy in patients with CML. The BCR-ABL1 ratio is defined as $\frac{[{{{\rm{PBLC}}}}]}{2[{{{\rm{PBHC}}}}]+[{{{\rm{PBLC}}}}]}$, as described in Clapp et al.^72^, where [PBLC] and [PBHC] represent the counts of leukemia cells and hematological cells in the peripheral blood, respectively. The dots show the clinical data obtained from Clapp et al.^72^, in which the patients responded well to imatinib. **c** The dynamics of the PBLC ratio (*R*_PBLC_) for 10 virtual patients who received continuous imatinib therapy. The therapy was initiated randomly for each patient after the PBLC ratio (*R*_PBLC_) reached a level between 5% and 25%. The triangle markers indicate the start of TKI therapy, while the dots at the end of some curves show the death of those patients. **d** The dynamics of the TME index. Data were obtained from the group of 10 virtual patients. **e** The percentage of CML patients achieving complete molecular remission varies based on their PBLC ratio at treatment initiation, noted by *R*_start_. **f** The relationship between the PBLC ratio and TME index during CML progression before and after continuous imatinib treatment. Data were obtained from the group of 10 virtual patients. All parameter values are the same as in Supplementary Table 1. Initial values of [HSPC] and [PBHC] are set to 1.8 × 10^6^ and 1.44 × 10^7^cells, respectively, and the values of [LSPC], [PBLC], and *Q* are set to zero.

Before 1975, clinical data indicated that the survival rate of CML patients was very low. However, since 2021, there has been a significant improvement in survival rate due to the use of tyrosine kinase inhibitor (TKI) therapy^60^. A randomized CML study by the German CML study group with a long-term evaluation of imatinib showed that the 10-year overall survival had reached 85–90%^61,62^. To model the progression of CML after imatinib therapy, several effects of imatinib on the leukemia cells were assumed in the current study. These effects include a decrease in the proliferation rate of LSPCs, an increase in the apoptotic rate of LSPCs, and a decrease in the amplification rate from LSPCs to PBLCs. Model parameters were then adjusted to reproduce the long-term overall survival after continuous TKI treatment (Fig. 3a). Additionally, the parameters were fine-tuned to replicate the clinical data depicting the rapid decrease of BCR-ABL1 ratios in peripheral blood post-TKI treatment administration (Fig. 3b). Virtual patients were first generated in simulations based on the overall survival curve from clinical data without treatment. Then, it was assumed that each patient was diagnosed and started TKI therapy during the chronic or accelerated phases when the ratio of leukemia cells in peripheral blood (*R*_PBLC_) is 5−25%.

Figure 3c shows the time evolution of the ratio of leukemia cells in the peripheral blood of 10 virtual patients who received continuous TKI treatment. The results showed that the ratio of leukemia cells continuously decreased in most patients, leading to complete molecular remission where the leukemia cell ratio becomes undetectable. However, a few patients receiving late treatment may still develop to death. Figure 3d shows the evolution of the TME index of the patients. Following TKI treatment, the TME index continued to rise before eventually decreasing to a low value, indicating a normal microenvironment after continuous treatment.

Simulations showed that the timing of TKI treatment initiation significantly influenced the changes in achieving complete molecular remission. Those patients diagnosed and treated earlier, when the PBLC ratio is low, have a much higher probability of long-term survival. On the other hand, patients diagnosed in the blast crisis phase, where the PBLC ratio is greater than 20%, experience a significant decline in the survival rate (Fig. 3e).

The combination of PBLC ratio (*R*_PBLC_) and the TME index provided insights into the progression of CML to complete molecular remission after imatinib therapy (Fig. 3f). As the recovery process began, *R*_PBLC_ rapidly decreased from the blast crisis (BC) phase towards the accelerated phase (AP), while the increasing rate of the TME index slowed down (also referred to Fig. 3d). In the chronic phase (CP), *R*_PBLC_ was typically very low (*R*_PBLC_ < 1%), and the TME index decreased toward the normal microenvironment, while *R*_PBLC_ slowly decreased towards complete remission. It is worth noting that the TME index can differ for the same level of PBLC ratio in the two processes during the CP, indicating a distinction between CML progression and the remission process. This prompted us to examine whether microenvironment conditions played a crucial role in treatment-free remission after discontinuing imatinib.

### CML progression after imatinib discontinuation

**Fig. 4:**
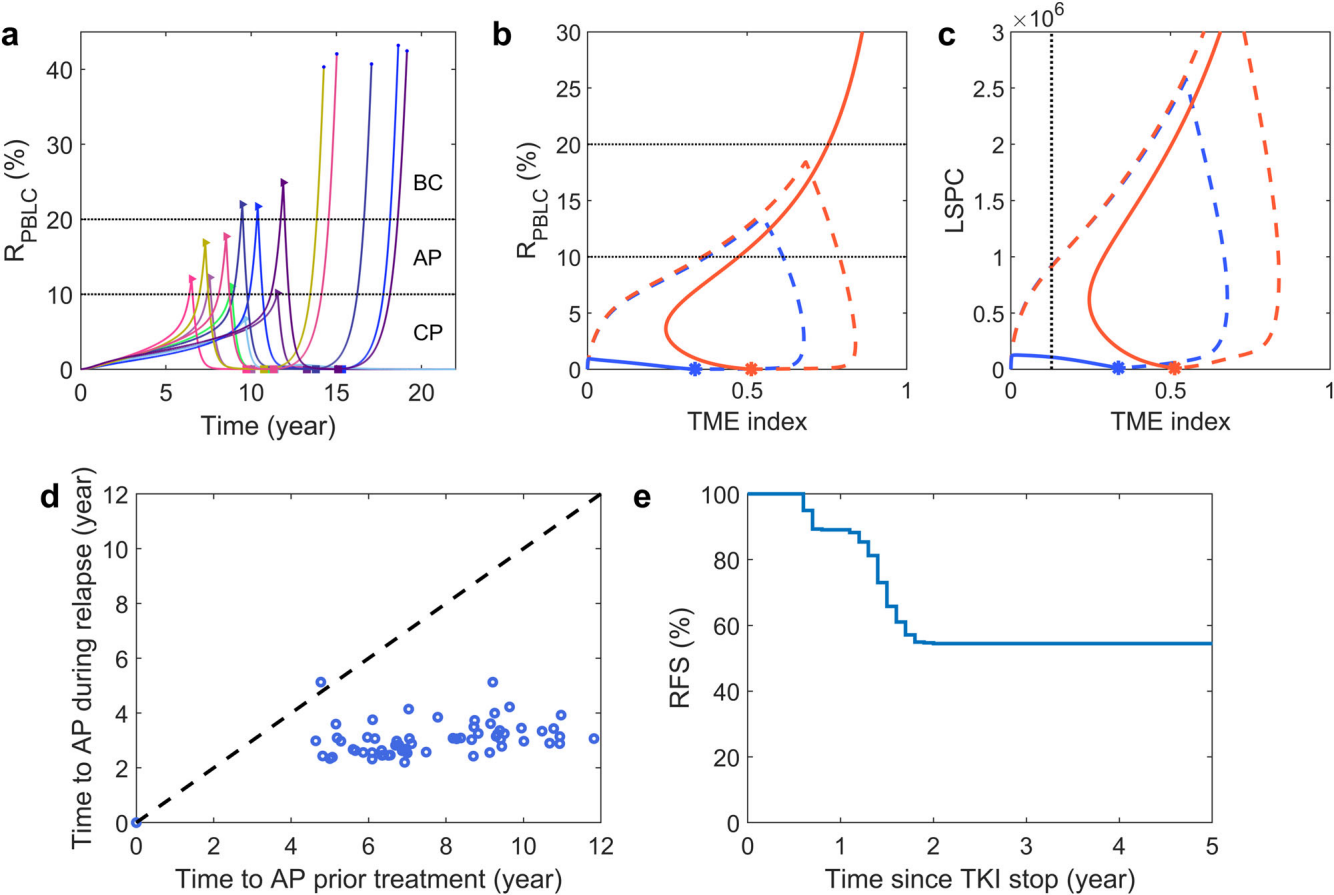
CML evolution for patients with discontinuous TKI therapy. **a** Dynamics of PBLC ratio (*R*_PBLC_) for virtual patients with TKI therapy initiated randomly when 5% < *R*_PBLC_ < 25% and stopped when *R*_PBLC_ = 0.01%. The starting and stopping points of TKI therapy are marked with triangle and square markers, respectively. The circle points at the end of the curves indicate the death of the virtual patients. **b** Typical trajectories of PBLC ratio versus TME index for two patients with relapse (red) and TFR (blue) after treatment discontinuation. The trajectories before and after treatment discontinuation are shown with dashed and solid lines, respectively. The stars indicate the points of treatment discontinuation. **c** Trajectories of LSPC versus TME index for the two patients in (**b**) with relapse (red) and TFR (blue). The vertical black dashed line shows the critical line (*Q* = 0.127) of the TME index that separates the fate of either tumor relapse or TFR. The critical value is discussed in Methods. **d** Comparison of the time to AP during phases of prior treatment and during the relapse process after stopping treatment in each relapsed patient. **e** Molecular relapse-free survival (RFS) curve after TKI stop. All parameter values are the same as in Supplementary Table 1.

We further investigated the progression of CML after discontinuing imatinib treatment. Following the above simulation of imatinib therapy, we stopped the treatment when the peripheral blood leukemia cell (PBLC) ratio reached 0.01% (*R*_stop_ = 0.01%) and continued to model the progression of the disease for a minimum of 15 years. Our simulation results, presented in Fig. 4a, revealed that some virtual patients experienced a recurrence of leukemia cells soon after discontinuing TKI therapy. However, others showed long-term treatment-free remission (TFR) with an extremely low PBLC ratio in peripheral blood.

In Fig. 4b, c, we showed the typical dynamics for virtual patients who relapsed and those who achieved treatment-free remission (TFR). Both cases showed very low PBLC ratios and LSPC numbers at treatment discontinuation (indicated by stars). However, the relapsed patient (shown in red) exhibited an increase in *R*_PBLC_ and a decrease in the TME index shortly after treatment stopped, followed by a quick increase in *R*_PBLC_ and the TME index, leading to irreversible AP. On the other hand, the TFR patient (shown in blue) demonstrated a continuous decrease in the TME index. Initially, the *R*_PBLC_ was increased, followed by a rapid decline towards a state of TFR.

Based on the relationship between the TME index and LSPC levels, we defined a critical line (indicated by the black dashed line in Fig. 4c) that can determine whether a patient will experience tumor relapse or TFR. This critical line helps in predicting the outcomes of treatment discontinuation. If a patient’s trajectory passes this line, it indicates a decrease in the LSPC number and TME index, leading to TFR. On the other hand, if the patient’s trajectory does not cross this line, it indicates an increase in the LSPC number and TME index, leading to the tumor relapsed (Fig. 4c). For more detailed information, please refer to the discussions in “Methods”.

We compared the periods required for developing into AP during the early stage of the disease progression and the relapse stages of each relapsed patient. Our analysis revealed that the periods in the relapse process were much shorter than those in the early progression stage. As per our findings, the relapse process typically progressed to AP in less than six years, as can be seen in Fig. 4d. This outcome is indicative of the fact that the relapse process progresses at a faster pace compared to the early stage of the disease.

The dynamics described above suggested that a PBLC ratio threshold of *R*_PBLC_ > 10% could serve as a criterion for detecting leukemia cell recurrence. By using this threshold, we generated the molecular relapse-free survival (RFS) curve for a cohort of 2000 virtual patients who stopped treatment at MR4.0 (PBLC ratio *R*_PBLC_ < 0.01%) (Fig. 4e). In our analysis, molecular relapse was identified at *R*_PBLC_ > 1% and confirmed when *R*_PBLC_ > 10%. We defined the timing of molecular relapse as the point when *R*_PBLC_ reaches 1%, and there was a subsequent time lag of approximately 5 months for RFS to decrease after treatment cessation. The simulation results aligned with clinical trials, where nearly 43% of patients rapidly experienced molecular recurrence upon discontinuing the treatment^16–18,20,21^. The molecular relapse-free survival curve displayed a clear plateau, indicating a positive long-term TFR outcome. Therefore, the findings from both clinical studies and modeling simulations supported the existence of two distinct patient groups: those prone to early molecular relapse and those capable of achieving TFR after TKI discontinuation. It has been observed that the RFS curve takes around 2 years to reach a plateau. However, clinical data shows that most relapses occur within 6 months after stopping the treatment. This suggests that other mechanisms not included in the current model might be at play, accelerating relapse progression.

### Mechanisms of treatment-free remission after imatinib discontinuation

**Fig. 5:**
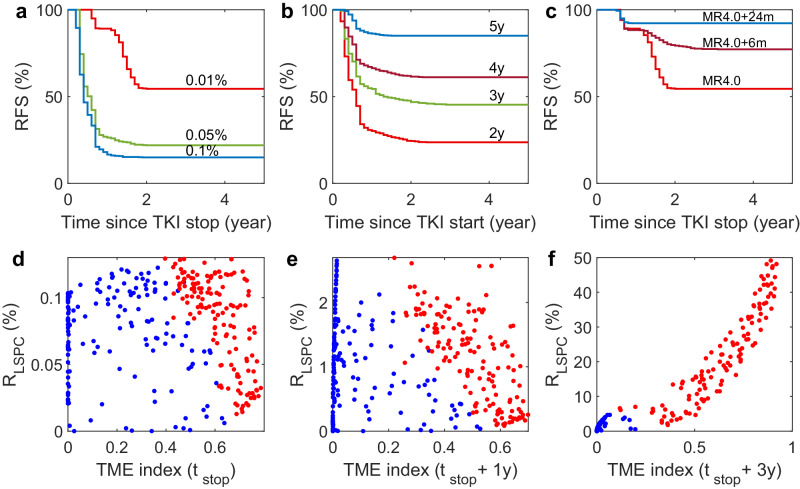
Mechanisms of TFR after imatinib discontinuation for virtual patients. **a** Molecular relapse-free survival (RFS) curves of virtual patients stopped imatinib therapy at PBLC percentage level *R*_PBLC_ = 0.01%, 0.05%, 0.1%, respectively. **b** Molecular relapse-free survival curves for virtual patients who stopped imatinib therapy after treatment for 2, 3, 4, and 5 years, respectively. **c** Molecular relapse-free survival curves of virtual patients who stopped imatinib therapy when PBLC percentage level reaches MR4.0 (*R*_PBLC_ = 0.01%), or with 6 or 24 more months of treatment after *R*_PBLC_ = 0.01%. **d**–**f** LSPC percentage and TME index at the time of stopping treatment, 1 and 3 years after stopping treatment for virtual patients, respectively. Treatment is stopped when the PBLC percentage level reaches *R*_PBLC_ = 0.01%. Red dots represent relapsed patients, and blue dots represent TFR patients after treatment discontinuations.

We conducted a study to examine the factors associated with molecular relapse following the discontinuation of imatinib. To do so, we analyzed the virtual patient responses under various conditions before the treatment was stopped. Our findings suggested that the PBLC ratio at the time of treatment stop (*R*_stop_) is a crucial factor in determining molecular relapse-free survival. Higher levels of the ratio *R*_stop_ increased the potential for early molecular relapse while decreasing the probability of treatment-free remission (TFR) (Fig. 5a).

In addition, the duration of imatinib treatment after diagnosis or the duration of deep molecular response (DMR) (MR4.0 or lower) before imatinib discontinuation is closely related to the probability of TFR^15^. Our model simulations showed that stopping imatinib treatment too early (within 3 years) can result in a high relapse probability (over 50%) and should be avoided. However, the probability of cumulative relapse can be reduced to 40% after 4 years of treatment and 10% after 5 years of treatment (Fig. 5b).

Furthermore, our study indicates that prolonging the duration of DMR is linked with an increased likelihood of TFR. Extending the DMR for one more year before discontinuing imatinib treatment increases the probability of achieving TFR to above 90% (Fig. 5c). However, the model simulation did not replicate the clinical data, as the EUROSKI study found that an additional year of DMR only increased the TFR rate by 2–3%^63^. One possible explanation for this discrepancy is the potential decay in drug efficiency during the later stage of treatment. Nevertheless, we did not detail this issue due to the deficiency of evidence and left it for future studies.

To study the mechanisms of TFR after discontinuing imatinib, we divided virtual patients into two groups—relapsed or long-term TFR—and analyzed the combination of bone marrow leukemia cell ratio and the TME index at the time of treatment cessation. It’s important to note that many TFR patients still have residual leukemia cells in their bone marrow. We found that the TME index was generally higher in relapsed patients than in TFR patients (Fig. 5d). After treatment discontinuation, both relapsed patients and TFR patients experienced an increase in bone marrow leukemia cell ratio and a decrease in TME index during the first year (Fig. 5d, e). However, in the long term (3 years after treatment discontinuation), relapsed patients experienced an increase in both bone marrow leukemia cell ratio and TME index, while TFR patients maintain low leukemia cell ratios (*R*_LSPC_ < 5%) in the bone marrow and small TME indices (Fig. 5f).

Our model includes a time-dependent function called *η*(*t*), which represents external factors that could affect the production of leukemia cells from normal HSPCs. This function is essential in modeling the initiation of leukemia cells and could also influence the recurrence rate of leukemia after treatment withdrawal. To avoid the possibility that mutations could induce tumor relapse, we have selected a specific pattern of mutation frequency that makes the factor *η*(*t*) extremely low at the time of treatment withdrawal. Thus, leukemia recurrence or TFR after treatment withdrawal depends not on external factors but on the interactions between microenvironment conditions and leukemia cells.

**Fig. 6:**
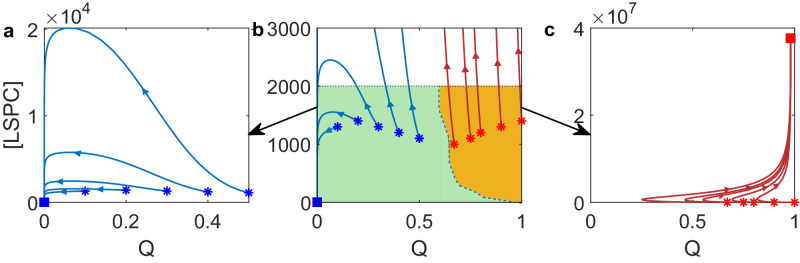
Phase space for the ordinary differential equation model (3). **a** Solutions with a small initial TME index converge to the stable steady state without LSPC. **b** The separation of regions of initial values of [LSPC] and *Q* that either developed to TFR (green) and tumor relapse (orange). Here, the initial value [HSPC] = 1.0 × 10^7^. **c** Solutions with a large TME index converge to a steady state with a high level of LSPC.

To gain a better understanding of the different responses observed after treatment discontinuation, we used a simplified ordinary differential equation model that considers only HSPC, LSPC, and the TME index (*Q*) (see equation (3) in “Methods”). This model describes the interaction between hematopoietic stem progenitor cells and the microenvironment. We studied the distinct responses after treatment discontinuation at MR4.0, corresponding to the bistability of two steady states. We set the initial conditions at [HSPC] = 1.0 × 10^7^ and [LSPC] < 2000, so that the BCR-ABL1 ratio $\frac{[{{{\rm{LSPC}}}}]}{2[{{{\rm{HSPC}}}}]+[{{{\rm{LSPC}}}}]}$ is less than 0.01%. We varied *Q* from 0 to 1 and used this initial condition to solve equation (3). The results showed that solutions with a small initial TME index tended to converge to a steady state without LSPC (Fig. 6a), which correspond to the situation of TFR, while solutions with a large initial TME index converge to a steady state with a high level of LSPC (Fig. 6c), which indicate the situation of tumor relapse. Figure 6b shows regions of initial values of [LSPC] and the TME index that either developed to TFR (green) or tumor relapse (orange). These results reveal the different responses after treatment discontinuation depend on the TME index at treatment discontinuation.

### Prediction of treatment-free remission

**Fig. 7:**
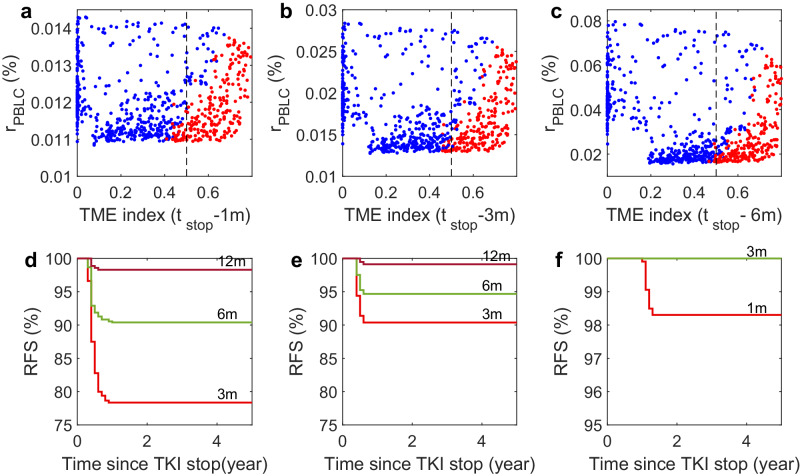
Prediction of treatment-free remission for virtual patients. **a**–**c** TME index and *R*_PBLC_ for TFR patients (blue dots) and relapsed patients (red dots) at (**a**) 1 month, (**b**) 3 months, and (**c**) 6 months before stopping the imatinib treatment. Dashed lines indicate the separation with TME index *Q* = 0.5. **d** Molecular relapse-free survival (RFS) curves for patients who stopped treatment at 3 months, 6 months, or 12 months after satisfying the predictive criterion P1. **e** RFS curves for patients who stopped treatment at 3 months, 6 months, or 12 months after satisfying the predictive criterion P2. **f** Molecular relapse-free survival (RFS) curves for patients who stopped treatment at 1 or 3 months after satisfying the predictive criterion P3.

We conducted a study to determine the factors predicting treatment-free remission (TFR) after stopping imatinib treatment. We analyzed the ratio of leukemia cells in peripheral blood (*R*_PBLC_) and the tumor microenvironment (TME) index in two groups of virtual patients at 1, 3, and 6 months before stopping imatinib treatment (Fig. 7a–c). Our findings showed that the TME indexes can effectively distinguish between the two groups of virtual patients. Those who achieved TFR had the values of TME index less than 0.5 at 6 months before stopping the treatment, and the value of *R*_PBLC_ decreased towards the stopping condition of MR4.0 (*R*_PBLC_ < 0.01).

The above observations suggested that the combination of PBLC ratio and TME index can be used to predict the probability of TFR in patients who stop imatinib treatment. To do this, we examined different predictors based on the values of *R*_PBLC_ and the TME index before treatment was stopped. These predictive criteria are classified as follows:

- P1: *R*_PBLC_ < 0.05%, TME index < 0.5;
- P2: *R*_PBLC_ < 0.05%, TME index < 0.4;
- P3: *R*_PBLC_ < 0.01%, TME index < 0.5.

We analyzed the molecular relapse-free survival curves for patients who stopped treatment after satisfying P1 or P2. The results showed that 78% of patients who stopped treatment 3 months after satisfying P1 achieved TFR (Fig. 7d). This is higher than that achieved TFR under the stopping condition of MR4.0, as shown in Fig. 4e. If a more strict condition like P2 is applied, the probability of TFR can increase to 90% (Fig. 7e). The RFS showed no significant difference for conditions P1 and P2 if the treatment was stopped 6 months or later after the conditions were met, as shown in Fig. 7d, e. However, if a more stringent criterion like P3 was applied, it was found that about 98% of patients could achieve TFR if the treatment were stopped at 1 month later, and 100% of patients could achieve TFR if the treatment were stopped at 3 months after the condition P3 was met (Fig. 7f). Based on model simulations, these results shed light on how the combination of PBLC ratio and TME index can help predict the outcome of TFR after treatment discontinuation.

### Treatment-free remission related parameters

**Fig. 8:**
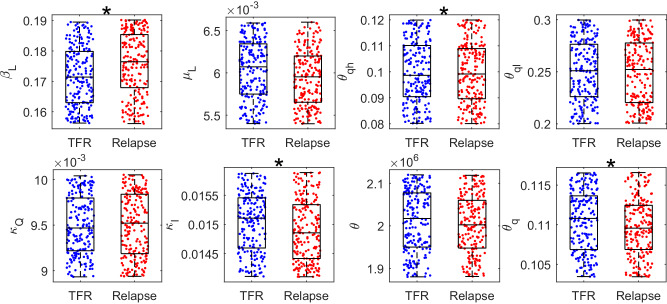
Distribution of parameter values for relapsed and TFR patients. The parameter value distributions of *β*_*L*_, *μ*_*L*_, *θ*_*qh*_, *θ*_*ql*_, *κ*_*Q*_, *κ*_*I*_, *θ*, and *θ*_*q*_ for relapsed patients (red) and TFR patients (blue). Black starts indicate the parameters with *p*-values less than 0.05 (*t*-test). Here, *β*_*L*_ and *μ*_*L*_ represent the proliferation rate and apoptosis of LSPC, respectively; *θ*_*qh*_ and *θ*_*ql*_ indicate the effective TME level for promotion of HSPC apoptosis and inhibition of LSPC apoptosis, respectively; *κ*_*Q*_ and *κ*_*I*_ imply the rates of transformation from NME to TME, and rates of restoring NME from TME, respectively; *θ* represents the effective LSPC level in promoting NME to TME transition; *θ*_*q*_ indicates effective TME level in inhibition of TME depredation.

In this section, we discussed the parameters related to TFR responses. We analyzed the parameters for two groups of patients: those who experienced early relapse and those who achieved TFR after treatment discontinuation (as shown in Fig. 8). Here, we mainly focused on the parameters associated with leukemia cell proliferation and apoptosis and the regulation of TME. Our findings indicated that the two groups have distinct preferences in terms of the leukemia cell proliferation rate (*β*_L_) and the repression of TME to HSPC survival (*θ*_*qh*_). Patients who relapsed tend to have a larger value of LSPC proliferation rate and a lower survival rate of HSPC under tumor microenvironment. Moreover, we observed that the parameters associated with the transition between normal and tumor microenvironments, namely *κ*_*I*_ and *θ*_*q*_, showed different performances for the two groups of patients. TFR patients have higher rates of restoring NME from TME, *κ*_*I*_, and a highly effective TME in inhibiting TME depletion, *θ*_*q*_. There was no significant difference in other parameters shown in Fig. 8.

We conducted a sensitivity analysis of the primary dynamic parameters for the two groups of patients to investigate the parameter dependences further. For the group of patients who underwent TFR, the duration of continuous TKI treatment required to achieve MR4.0 is strongly and positively correlated with the transition rate from normal to tumor microenvironment (*κ*_*Q*_). In contrast, it is negatively correlated with the apoptosis rate of LSPC (*μ*_L_), as well as parameters associated with TME regulation (*θ*_*q*_, *θ*_*ql*_, and *θ*) (Supplementary Fig. 5a). For the group of relapsed patients, the survival time since TKI treatment is positively linked with the promotion of TME to leukemia cell survival (*θ*_*ql*_ and *μ*_L_) and negatively linked with the proliferation rate of LSPCs (*β*_L_) and the transition rate from normal to the tumor microenvironment (*κ*_Q_) (Supplementary Fig. 5b).

### Second attempt at treatment-free remission

**Fig. 9:**
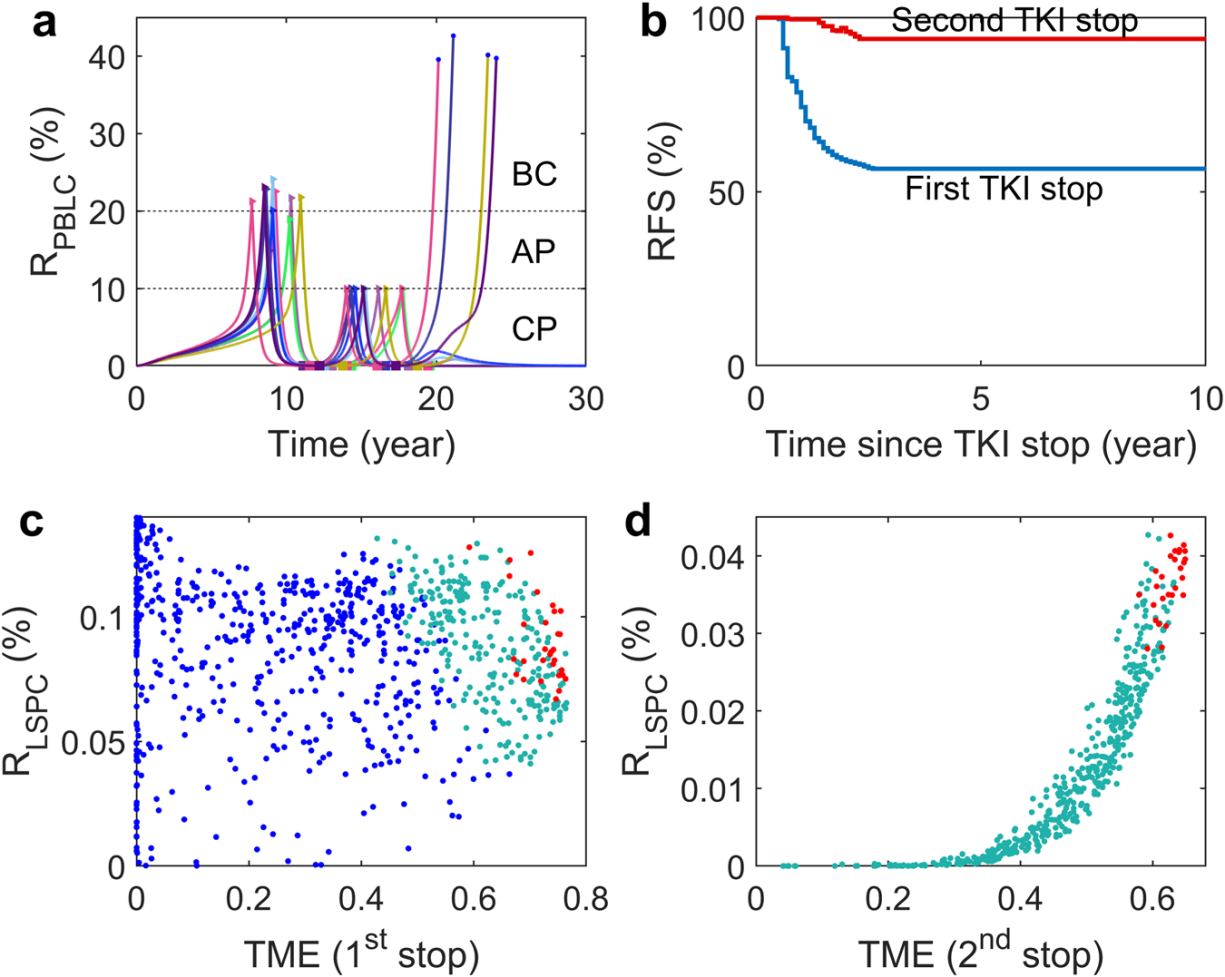
CML evolution and mechanisms of TFR after the second discontinuation of imatinib treatment in virtual patients. **a** Dynamics of PBLC ratio (*R*_PBLC_) for 10 virtual patients. **b** Molecular relapse-free survival curves of patients. **c** LSPC percentage and TME index when stopping imatinib treatment for the first time. Blue dots represent the data for TFR patients after the first imatinib discontinuation; cyan dots represent patients with CML relapse after the first imatinib discontinuation but TFR after the second imatinib discontinuation; red dots represent relapsed patients after both the first and second imatinib discontinuations. **d** LSPC percentage and TME index when stopping imatinib treatment for the second time. Cyan dots represent the data for TFR patients after the second imatinib discontinuation; red dots represent relapsed patients after the second imatinib discontinuation. In all the simulations, virtual patients started imatinib treatment randomly when 5% < *R*_PBLC_ < 25%, stopped imatinib treatment at *R*_stop_ = 0.01% of PBLC percentage level for the first time, restarted treatment when *R*_PBLC_ ≥ 10%, and then stopped imatinib therapy again at *R*_stop_ = 0.01% of PBLC percentage level.

We further investigated the chances of achieving TFR in patients who experienced molecular recurrence after their first TKI discontinuation. To do this, we resumed treatment when *R*_PBLC_ was equal to or greater than 10% and then stopped it again at MR4.0. The time evolution of *R*_PBLC_ is shown in Fig. 9a. During the second TKI treatment, some patients who relapsed after the first TKI discontinuation can achieve TFR after stopping the treatment at the second attempt, as indicated by the cyan dots in Fig. 9c, d. This result confirmed that failing the first TKI discontinuation attempt does not necessarily indicate failure of a second TKI discontinuation, as observed in clinical trials^27,64^. However, some patients with a high value of TME index at both the first and second treatment stops relapsed again after the second TKI discontinuation (shown as red dots in Fig. 9c, d).

Model simulations showed that the molecular relapse-free survival rate was significantly higher after the second treatment was stopped than after the first attempt (see Fig. 9b). This result is reasonable because the patients who failed after the first TFR attempt received the second TKI treatment when *R*_PBLC_ reached 10%, earlier than most newly diagnosed patients. As seen in Fig. 9d, many patients who achieved TFR after the second stopping treatment have a low value of the TME index. Our simulation showed that the TFR rate increased from 57% after the first TKI discontinuation to about 90% after the second TKI discontinuation.

However, clinical reports from the French RE-STIM study suggested that there was no significant difference in the TFR rates between the first and the second treatment discontinuation^27^. Clinical trials suggested that the most important factors for estimating the second TFR success are the speed of molecular relapse and the TKI-free duration after the first TKI discontinuation attempt^27^. The discrepancy between modeling simulation and clinical studies suggests that other factors that may affect the success of the second TFR attempt were not considered in the current study. Moreover, although there are many studies and guidelines on the first TFR, only very few studies on the second TFR^65^. More data is still needed to understand better the second TFR attempts in patients with CML.

### Importance of tumor microenvironment dynamics

The current study proposed that the interaction between leukemia stem cells and the tumor microenvironment (TME) is a crucial factor in determining patient outcomes after discontinuing imatinib treatment. We found that the positive feedback loop between leukemia cells and TME is responsible for the different responses between early relapse and long-term treatment-free remission (TFR). To determine the necessity of this feedback loop, we conducted further investigations by modifying the model to assume a constant TME index. We then examined how the virtual patients responded after discontinuing treatment when subjected to this constant TME index. The results, presented in Supplementary Fig. 6, showed the time courses of PBLC ratios for patients with constant TME indices and treatment discontinued at MR4.0 (PBLC ratio *R*_PBLC_ < 0.01%).

When the TME index equaled 0 or 0.05, most patients remained in the chronic phase (CP) and did not require any treatment. For the minority of patients who did receive treatment, more patients did not have molecular recurrence after treatment was stopped, as shown in Supplementary Fig. 6a, b. However, as the TME index increased to 0.1 and 0.15, the percentage of patients who progress to the accelerated (AP) and blast crisis (BC) phases after treatment discontinuation obviously increased (Supplementary Fig. 6c, d). When the TME index was even higher, at 0.2 or 0.5, all patients might progress to the BC phase after treatment discontinuation (Supplementary Fig. 6e, f). Therefore, maintaining the TME below 0.15 was necessary to achieve TFR after treatment discontinuation if we assumed a constant TME.

**Fig. 10:**
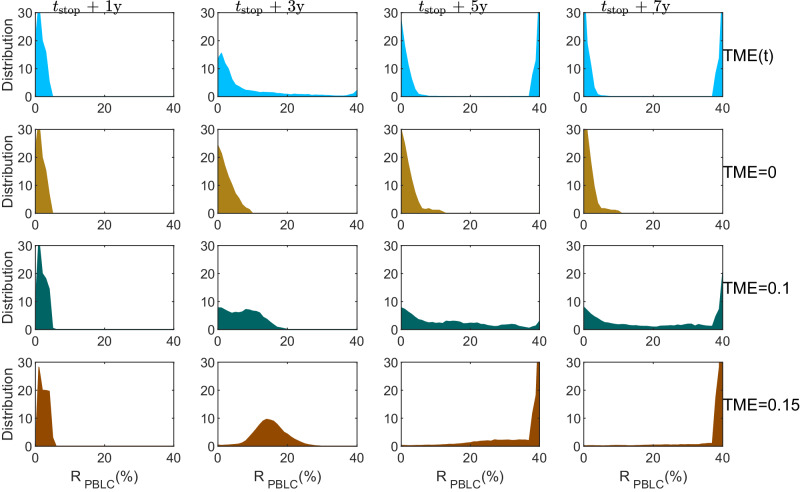
Evolution of PBLC ratio distribution after TKI treatment discontinuation for virtual patients. Four-row panels show the distributions of PBLC ratios at 1, 3, 5, and 7 years after TKI treatment discontinuation for virtual patients with dynamically changed TME based on the proposed random differential equation model (TME(*t*)), or constant TME with TME = 0, 0.1, and 0.15, respectively. Here, *t*_stop_ indicates the time point of TKI discontinuation. The mortalities of virtual patients were ignored in model simulations. The distributions are calculated from 1000 samples for each case.

Figure 10 displays the distributions of PBLC ratio (*R*_PBLC_) from 1 to 7 years after treatment discontinuation. The results demonstrated that when TME changes dynamically, there can be a bimodal distribution of *R*_PBLC_ five years after treatment discontinuation. On the other hand, a constant TME can only result in an unimodal distribution in *R*_PBLC_. These findings suggested that the interaction between the microenvironment and leukemia stem cells, which brings about dynamic changes in TME, is crucial for the dual responses observed after treatment discontinuation.

To better understand how CML evolves under different TME conditions, we analyzed the time it takes for patients to reach the accelerated phase (AP) and blast crisis (BC) stages since the appearance of BCR-ABL1 cells. We found that patients with dynamically changing TME took varying amounts of time to reach AP, with the range falling between those for patients with TME = 0.05 and TME = 0.1 (Supplementary Fig. 7a). On the other hand, the time taken for patients to reach the BC phase, with dynamically changing TME, was similar to that for patients with TME = 0.1 (Supplementary Fig. 7a). Based on these results, we conclude that a TME value less than 0.1 is necessary for a reasonable replication of the progression of CML evolution.

In addition, we examined the survival curve of virtual patients who received continuous treatment. Our findings revealed that the model could only replicate an 80% survival rate if the constant TME index was as high as 0.95, as shown in Supplementary Fig. 7b. However, we observed that when TME exceeded 0.5, all patients experienced molecular recurrence after the withdrawal of TKI (Supplementary Fig. 6f), which contradicts experimental observations. Therefore, we concluded that the constant TME index is unlikely to reproduce CML evolution dynamics for both the treatment discontinuation and continuous-treatment scenarios. As a result, a dynamically changing TME index is essential for CML evolution.

## Discussion

Chronic myeloid leukemia (CML) is a rare form of cancer that heavily relies on a single genetic mutation. Tyrosine-kinase inhibitors (TKIs) have been crucial in treating patients with CML. Many patients receive a deep molecular response after five or more years of treatment. Recently, it has become common to recommend discontinuing TKI treatment to achieve treatment-free remission (TFR). Clinical studies have shown that roughly 50% of patients with sustained deep molecular response can discontinue TKI treatment and remain in TFR. However, most relapses occur within the first six months. For those who do relapse, most of them can regain major molecular responses by resuming the original TKI treatment, and some can even achieve a second TFR after the first attempt failed. Nonetheless, the factors determining the success of TFR are not well understood, and research is ongoing to identify predictors.

Although TKI treatment can significantly reduce leukemia cells, it may not eliminate all of them^66^. Recent studies suggested that CML stem cells can persist in some patients even after TKI discontinuation^28^. Patients who have few residual leukemia cells after discontinuing TKI treatment may experience either early relapse or long-term TFR, leading to a dichotomy in outcomes. The tumor microenvironment (TME) may be critical in regulating the production and survival of progenitor cells, affecting the success of TFR.

A stochastic mathematical model was developed in the current study to investigate CML progression after TKI discontinuation. The model was designed to explore the dynamics of CML progression with and without imatinib therapy. We validated the model against the three CML evolution phases and clinical overall survival curves. The model predicted the dichotomous response of patients to imatinib discontinuation, which is consistent with clinical observations. Additionally, the model highlighted the significance of the TME index, which changes dynamically due to feedback between leukemia stem cells and the microenvironment. The TME index is important for understanding the two possible outcomes of patients’ response to imatinib discontinuation.

Based on model simulations, we proposed that a combination of the ratio of leukemia cells in peripheral blood (PBLC) and the TME index can be useful in predicting patients with TFR after the treatment is stopped (Fig. 7). However, it is important to note that the TME index used in the model is a theoretical value that does not correspond to any specific molecular mechanisms. Our purpose is to differentiate between normal and tumor microenvironments, and it cannot be directly linked to clinical measurements. Several factors in the microenvironment have been associated with the disease progression. For instance, physiologic hypoxia supports the maintenance of CML stem cells^67^. Additionally, modifying the bone marrow microenvironment through osteoblastic cell-specific activation of the parathyroid hormone (PTH) receptor can attenuate BCR-ABL1-induced CML-like myeloproliferative neoplasia^68^. Measuring the condition of the microenvironment as the disease progresses remains a challenging task.

Our study conducted an attempt to explore TFR in CML patients through a mechanism-driven mathematical model. Unlike previous mathematical modeling studies that only considered the leukemia cell population dynamics^40–42,44–47^, we incorporated the interaction between leukemia cells and the microenvironment. Our novel findings highlight the significance of the interaction between tumor cells and the microenvironment. They suggest that a normal microenvironment can maintain the state of TFR, even when minimal residual leukemic cells are present. Additionally, the positive feedback between leukemia cells and the tumor microenvironment is an important mechanism that explains the two distinct patient groups: those prone to early molecular relapse and those capable of achieving TFR after TKI discontinuation. In contrast, previous population dynamical models indicate that all patients would relapse after stopping the TKI treatment^44,46,47^.

Moreover, the relationship between TME and physiological indices in a clinical setting requires further investigation. In summary, our study highlights the importance of the microenvironment in TFR. Effective methods to measure and control the microenvironment can play a crucial role in improving cancer therapy and achieving the final goal of TFR.

At its current stage, the model is primarily conceptual and requires further refinement to incorporate additional details. These potential enhancements include addressing the heterogeneity of leukemic stem cells, considering mutations that can lead to drug resistance, accounting for external perturbations affecting the microenvironment, and providing more detailed descriptions of the microenvironment.

There are two issues with the current model that need further consideration. First, our proposed model has a drawback related to delay. Clinical data indicates that most relapses occur within the first six months of therapy discontinuation. However, in our model, it takes around two years for the molecular relapse-free survival (RFS) curve to reach a plateau (Fig. 4e). This suggests that there may be additional, possibly unidentified, factors that contribute to the rapid relapse following treatment discontinuation.

Second, there is a drawback related to improving the RFS rate after prolonged DMR before treatment discontinuation. Our model simulations suggest that one more year of DMR before treatment stops can significantly increase the RFS rate. However, the EUROSKI study found that an additional year of DMR can only increase the RFS rate by 2 − 3%^63^. Therefore, extensive investigations are needed to better understand and address these aspects of the long-term dynamics of hematopoiesis in response treatment.

We have proposed a method of determining the disease age through CD34 expression, which is crucial in measuring the progression of the disease. However, it is important to note that the exact age of the disease is often unknown since the appearance of the first BCR-ABL1 cell is usually undetectable. Clinically, the disease age may not necessarily correspond with the elapsed time but instead with BCR-ABL1 ratio levels, which requires further investigation.

## Methods

### Mathematical model formulation

Based on the assumptions presented in Fig. 1, the process of chronic myeloid leukemia (CML) evolution can be described using the dynamics of the cell population numbers of hematopoietic stem progenitor cells ([HSPC]), leukemia stem progenitor cells ([LSPC]), hematopoietic cells in peripheral blood ([PBHC]), leukemia cells in peripheral blood ([PBLC]), and the tumor microenvironment (TME) index (represented by *Q*).

The proliferation of HSPC and LSPC is regulated by cytokines and intracellular signaling pathways. Despite the complexity of these pathways, a phenomenological formulation of Hill function dependence can be derived from simple assumptions regarding the interactions between signaling molecules and receptors^69,70^. Put simplicity, the proliferation rates of HSPC and LSPC decrease as the number of cells increases. These decreasing functions can be expressed as Hill-type functions $\frac{{\beta }_{{{{\rm{H}}}}}}{1+([{{{\rm{HSPC}}}}]+{\rho }_{1}[{{{\rm{LSPC}}}}])/{\theta }_{{{{\rm{H}}}}}}$ and $\frac{{\beta }_{{{{\rm{L}}}}}}{1+([{{{\rm{HSPC}}}}]+{\rho }_{2}[{{{\rm{LSPC}}}}])/{\theta }_{{{{\rm{L}}}}}}$, respectively. Here, *β*_H_ and *β*_L_ represent the maximum proliferation rates of HSPC and LSPC, respectively. The constants *θ*_H_ and *θ*_L_ represent the half-effective cell numbers that are related to the repression of proliferation pathways. Additionally, *ρ*_1_ and *ρ*_2_ denote the relative strength of growth inhibitors released by LSPC with respect to HSPC.

The apoptosis rates for different cells are denoted by *μ*_*X*_ (*X* = H, L, PH, PL). The differentiation rates of HSPC and LSPC from bone marrow to peripheral blood are denoted by *κ*_*X*_ (*X* = H, L). Before entering the peripheral compartment, both HSPC and LSPC go through a process of cell proliferation that amplifies their numbers. The rate of amplification rates are assumed to be *α*_*H*_ and *α*_*L*_, respectively.

The TME index *Q* is a numerical value ranging from 0 to 1. A higher value of *Q* means that tumor cells are more likely to survive, and the transition from normal to tumor microenvironment is controlled by tumor cells in the bone marrow. We hypothesized that leukemia cells might accelerate the shift from normal to tumor microenvironment, leading to an increase in the TME index with a rate ${\kappa }_{Q}\frac{{([{{{\rm{LSPC}}}}]/\theta )}^{n}}{1+{([{{{\rm{LSPC}}}}]/\theta )}^{n}}$. On the other hand, the tumor microenvironment can impede the transition from tumor to normal condition, causing the TME index to decline with a rate ${\kappa }_{I}\frac{Q}{1+{(Q/{\theta }_{q})}^{s}}$.

Cell survival depends on the microenvironment’s condition, and the apoptosis rate for LSPC decreases with an increasing TME index *Q*, expressed as $\frac{{\mu }_{{{{\rm{L}}}}}}{1+{(Q/{\theta }_{\mu })}^{2}}$. Additionally, we assumed that the tumor microenvironment hinders the survival of HSPC by promoting apoptosis at a rate ${\mu }_{0}\frac{{(Q/{\theta }_{qh})}^{2}}{1+{(Q/{\theta }_{qh})}^{2}}$.

To simulate the process of disease origination, we assumed that leukemia cells are randomly generated from normal HSPCs in healthy individuals. This was modeled using a Poisson random number, which is denoted by Ψ([HSPC], *η*) and is dependent on the rate of BCR-ABL1 fusion gene formation (*η*) and the number of normal cells [HSPC]. The expected number of LSPCs generated from HSPCs per day is given by

$${{{\rm{E}}}}[\Psi ]=\eta (t)\times [{{{\rm{HSPC}}}}](t),$$

where the time-dependent function *η*(*t*) takes into account the varying probability of fusion gene formation over time.

Our study also considered the impact of the tumor microenvironment (TME) on the creation and survival of cells with fusion genes. To achieve this, we used a model that combines the TME-induced promotion of leukemia cell formation, represented by *δ*(*Q*), and a phenomenological function, *η*_0_(*t*), which describes the time-dependent rate of fusion gene formation during disease progression. Thus, the time-dependent function *η*(*t*) is expressed as

$$\eta =\delta (Q)\times {\eta }_{0}(t).$$

The factors contributing to the formation of the BCR-ABL1 fusion gene are still not fully understood. It is known that abnormal stem cell niches, such as toxic microenvironments, are associated with the development of many cancers, including the persistence of cancer stem cells. To model the origin and progression of CML, it was assumed that microenvironmental conditions may be linked to the formation of leukemia cells. The promotion of leukemia cell formation by the TME is expressed by a Hill-type function as

$$\delta (Q)={\delta }_{L}\frac{{(Q/{\theta }_{a})}^{m}}{1+{(Q/{\theta }_{a})}^{m}}+{\varepsilon }_{0}.$$

The bifurcation analysis with constant mutation rate *η*_*Q*_ presented in Supplementary Fig. 3 suggests that if the value of *η*_0_ remains low, i.e., less than 10^−3^, normal stem cells will dominate the bone marrow, thereby preventing the progression of CML to the blast crisis (BC) stage. However, if the value of *η*_0_ is high, i.e., greater than 10^−3^, treatment-free remission (TFR) is not possible after stopping the treatment. Thus, to reproduce the whole process from disease initiation to TFR after stopping the treatment, the function *η*_0_(*t*) must vary with time so that *η*_0_(*t*) takes a high value during the onset of CML and decreases to a lower value at a later stage. To illustrate this, we assume that the value of *η*_0_ increases from 0 to a high level during the disease progression and eventually decreases to a lower level. We represented this requirement as a continuous function below:

$${\eta }_{0}(t)=\frac{1}{1+{e}^{-t/0.1}}\times \frac{1}{1+{e}^{-(t-{t}_{1})/{\tau }_{1}}}\times \frac{1}{1-{e}^{(t-{t}_{2})/{\tau }_{2}}}.$$

Here, *t*_1_ and *t*_2_ are constants used to control the timing of increasing and decreasing of the function *η*_0_. The parameters *τ*_1_ and *τ*_2_ regulate the shape of the function. The units of these constants are in years. Supplementary Fig. 1 shows the graph of the function *η*_0_(*t*), which indicates a pulse increasing at *t* = 0 followed by a decrease to zero at the later stage.

The feature of a pulse increasing in the function *η*_0_(*t*) is crucial in our study, while the mathematical formulation is insignificant. The underlying regulation mechanisms for the increasing and decreasing *η*_0_(*t*) are unknown biologically and can be highly diverse for different patients. Therefore, we only consider a time-dependent function *η*_0_(*t*) in our study, leaving the issue of modeling the production of leukemia cells for future considerations. In our study, we always set *t* = 0 as the timing of the rapidly increasing rate of fusion gene formation, which leads to the production of leukemia cells.

Based on the above assumptions, CML progression can be modeled using the following random differential equations (RDEs):

2 $$\left\{\begin{array}{ll}{{{\rm{d}}}}[{{{\rm{HSPC}}}}]\,=\,\left[\frac{{\beta }_{{{{\rm{H}}}}}}{1+([{{{\rm{HSPC}}}}]\,+\,{\rho }_{1}[{{{\rm{LSPC}}}}])/{\theta }_{{{{\rm{H}}}}}}-{\mu }_{{{{\rm{H}}}}}\left(1+{\mu }_{{{{\rm{0}}}}}\frac{{(Q/{\theta }_{qh})}^{2}}{1\,+\,{(Q/{\theta }_{qh})}^{2}}\right)-{\kappa }_{{{{\rm{H}}}}}\right]\\ \qquad\qquad\quad\times [{{{\rm{HSPC}}}}]{{{\rm{d}}}}t-\Psi ([{{{\rm{HSPC}}}}],\eta ){{{\rm{d}}}}t,\\ {{{\rm{d}}}}[{{{\rm{LSPC}}}}]\,=\,\left(\frac{{\beta }_{{{{\rm{L}}}}}}{1\,+\,([{{{\rm{HSPC}}}}]\,+\,{\rho }_{2}[{{{\rm{LSPC}}}}])/{\theta }_{{{{\rm{L}}}}}}-\frac{{\mu }_{{{{\rm{L}}}}}}{1\,+\,{(Q/{\theta }_{ql})}^{2}}-{\kappa }_{{{{\rm{L}}}}}\right)[{{{\rm{LSPC}}}}]{{{\rm{d}}}}t\\ \qquad\qquad\quad+\,\Psi ([{{{\rm{HSPC}}}}],\eta ){{{\rm{d}}}}t,\\ {{{\rm{d}}}}[{{{\rm{PBHC}}}}]\,=\,\left({\alpha }_{{{{\rm{H}}}}}{\kappa }_{{{{\rm{H}}}}}[{{{\rm{HSPC}}}}]-{\mu }_{{{{\rm{PH}}}}}[{{{\rm{PBHC}}}}]\right){{{\rm{d}}}}t,\\ {{{\rm{d}}}}[{{{\rm{PBLC}}}}]\,=\,\left({\alpha }_{{{{\rm{L}}}}}{\kappa }_{{{{\rm{L}}}}}[{{{\rm{LSPC}}}}]-{\mu }_{{{{\rm{PL}}}}}[{{{\rm{PBLC}}}}]\right){{{\rm{d}}}}t,\\ {{{\rm{d}}}}Q\,=\,\left({\kappa }_{Q}\frac{{([{{{\rm{LSPC}}}}]/\theta )}^{n}}{1\,+\,{([{{{\rm{LSPC}}}}]/\theta )}^{n}}(1-Q)-{\kappa }_{{{{\rm{I}}}}}\frac{Q}{1\,+{\,(Q/{\theta }_{q})}^{s}}\right){{{\rm{d}}}}t.\end{array}\right.$$

Furthermore, to better understand the mechanism of TFR, we simplified the above random differential equation model. This simplified model includes only the components of HSPC, LSPC, and the TME index (*Q*). We omit the fusion gene formation as it is irrelevant to our current focus on tumor cell recurrence independent of the formation of the BCR-ABL1 fusion gene. The resulting ordinary differential equation (ODE) model is as follows:

3 $$\left\{\begin{array}{lll}\frac{{{{\rm{d}}}}[{{{\rm{HSPC}}}}]}{{{{\rm{d}}}}t}\,=\,\left[\frac{{\beta }_{{{{\rm{H}}}}}}{1\,+\,([{{{\rm{HSPC}}}}]\,+\,{\rho }_{1}[{{{\rm{LSPC}}}}])/{\theta }_{{{{\rm{H}}}}}}-{\mu }_{{{{\rm{H}}}}}\left(1\,+\,{\mu }_{{{{\rm{0}}}}}\frac{{(Q/{\theta }_{qh})}^{2}}{1\,+\,{(Q/{\theta }_{qh})}^{2}}\right)-{\kappa }_{{{{\rm{H}}}}}\right]\\ \qquad\qquad\quad\times \,[{{{\rm{HSPC}}}}],\\ \frac{{{{\rm{d}}}}[{{{\rm{LSPC}}}}]}{{{{\rm{d}}}}t}\,=\,\left(\frac{{\beta }_{{{{\rm{L}}}}}}{1+([{{{\rm{HSPC}}}}]\,+\,{\rho }_{2}[{{{\rm{LSPC}}}}])/{\theta }_{{{{\rm{L}}}}}}-\frac{{\mu }_{{{{\rm{L}}}}}}{1\,+\,{(Q/{\theta }_{ql})}^{2}}-{\kappa }_{{{{\rm{L}}}}}\right)[{{{\rm{LSPC}}}}],\\ \qquad\frac{{{{\rm{d}}}}Q}{{{{\rm{d}}}}t}\,=\,{\kappa }_{Q}\frac{{([{{{\rm{LSPC}}}}]/\theta )}^{n}}{1\,+\,{([{{{\rm{LSPC}}}}]/\theta )}^{n}}(1-Q)-{\kappa }_{{{{\rm{I}}}}}\frac{Q}{1\,+\,{(Q/{\theta }_{q})}^{s}}.\end{array}\right.$$

This ODE model describes the interactions between hematopoietic stem progenitor cells and the microenvironment, which is essential in understanding the TFR phenomenon.

### Imatinib treatment

In the modeling of imatinib treatment, we did not consider the likelihood of drug resistance. It was assumed that imatinib would lead to a significant decrease in the proliferation rates of LSPCs and a significant increase in the apoptosis rate of LSPCs. Upon implementation of imatinib, it was assumed that the proliferation rate (*β*_L_) and the amplification rate (*α*_L_) of LSPCs would decrease to *β*_L_/*d*_1_ and *α*_L_/*d*_2_, respectively. Additionally, it was assumed that the apoptosis rate of LSPCs (*μ*_L_) would increase to *d*_3_*μ*_L_. Here, *d*_1_, *d*_2_, *d*_3_ > 1. Thus, we make the replacement

$${\beta }_{{{{\rm{L}}}}}\to {\beta }_{{{{\rm{L}}}}}/{d}_{1},\quad {\alpha }_{{{{\rm{L}}}}}\to {\alpha }_{{{{\rm{L}}}}}/{d}_{2},\quad {\mu }_{{{{\rm{L}}}}}\to {d}_{3}{\mu }_{{{{\rm{L}}}}}$$

in the equation (2) to demonstrate the effect of imatinib treatment.

### Data analysis

We conducted an analysis of the GEO database (GSE4170)^59^ to quantify the progression of CML evolution. This dataset contains gene expression data from 91 CML patients in three phases: chronic phase (with 42 cases), accelerated phase (with 17 cases), and blast crisis (with 32 cases). In CML, leukemia stem cells are characterized by CD34^+^ expression. A similarity score based on CD34^+^ expression has been proposed as a maker for CML progression^58^. As such, we compared CD34 expression levels across the three phases, which showed that the average CD34 expression level increases with CML progression (Fig. 2a). Therefore, we selected CD34 expression level as a marker to represent the disease age of CML from the emergence of BCR-ABL1 cells.

The exact disease age is usually unknown for the patients. An entropy-based method was proposed in Brehme et al.^58^ to estimate the days after mutation based on gene expressions. Here, we further simplify the estimation through the CD34 expression. To define the disease age for individual patients, we assumed a linear dependence between the CD34 expression level and the disease age. However, the coefficients for patients at different phases may vary. The disease age, denoted by *T*_disease age_ (measured in years), is calculated using the following formula:

4 $${T}_{{{{\rm{disease}}}}\,{{{\rm{age}}}}}=A\times [{{{\rm{CD}}}}34]+B.$$

Here, [CD34] represents the normalized CD34 expression level of a patient. Based on the research in Brehme et al.^58^, the coefficients (*A*, *B*) were chosen in such a way that the disease ages of patients at the chronic phase (CP), accelerated phase (AP), and blast crisis (BC) phases are distributed separately. Patients in CP have disease ages ranging from 2.5 to 5.5 years, while those in AP have disease ages ranging from 5.5 to 12.5 years, and patients in the BC phase have disease ages ranging from 7.5 to 14.3 years (Supplementary Fig. 2). Therefore, to represent the disease ages in each phase, the values of (*A*, *B*) were chosen as (5.0, 5.0) for CP patients, (4.5, 6.5) for AP patients, and (4.0, 10) for BC patients.

### Bifurcation analysis

The random formation of the BCR-ABL1 fusion gene and the tumor microenvironment (TME) changes are crucial factors in the progression of chronic myeloid leukemia (CML). To examine their effects on the long-term dynamics of CML progression, we conducted a bifurcation analysis based on the fusion gene’s production rate *η* and the TME index *Q*. We set *η* and *Q* as constants in equation (2) and replaced the random number Ψ([HSPC], *η*) with its expectation value, *η*[HSPC]. This enabled us to obtain a second-order ordinary differential equation for the cell numbers [HSPC] and [LSPC]. The equation is as follows:

5 $$\left\{\begin{array}{lll}\frac{{{{\rm{d}}}}[{{{\rm{HSPC}}}}]}{{{{\rm{d}}}}t}\,=\,\left[\frac{{\beta }_{{{{\rm{H}}}}}}{1\,+\,([{{{\rm{HSPC}}}}]\,+\,{\rho }_{1}[{{{\rm{LSPC}}}}])/{\theta }_{{{{\rm{H}}}}}}-{\mu }_{{{{\rm{H}}}}}\left(1\,+\,{\mu }_{{{{\rm{0}}}}}\frac{{(Q/{\theta }_{qh})}^{2}}{1\,+\,{(Q/{\theta }_{qh})}^{2}}\right)-{\kappa }_{{{{\rm{H}}}}}\right]\\ \qquad\qquad\times \,[{{{\rm{HSPC}}}}]-\eta [{{{\rm{HSPC}}}}],\\ \frac{{{{\rm{d}}}}[{{{\rm{LSPC}}}}]}{{{{\rm{d}}}}t}\,=\,\left(\frac{{\beta }_{{{{\rm{L}}}}}}{1\,+\,([{{{\rm{HSPC}}}}]+{\rho }_{2}[{{{\rm{LSPC}}}}])/{\theta }_{{{{\rm{L}}}}}}-\frac{{\mu }_{{{{\rm{L}}}}}}{1\,+\,{(Q/{\theta }_{ql})}^{2}}-{\kappa }_{{{{\rm{L}}}}}\right)[{{{\rm{LSPC}}}}]\\ \qquad\qquad+\,\eta [{{{\rm{HSPC}}}}].\end{array}\right.$$

We then performed a bifurcation analysis on the above equation with respect to *Q* and *η*.

From equation (5), when the fusion gene formation rate *η* is zero, there is a trivial steady-state *E*_0_ = (0, 0). The steady-state *E*_0_ is locally asymptotically stable if ${\beta }_{{{{\rm{H}}}}}\, < \,{\bar{\mu }}_{{{{\rm{H}}}}}:= {\mu }_{{{{\rm{H}}}}}\left(1+{\mu }_{{{{\rm{0}}}}}\frac{{(Q/{\theta }_{qh})}^{2}}{1+{(Q/{\theta }_{qh})}^{2}}\right)+{\kappa }_{{{{\rm{H}}}}}$ and ${\beta }_{{{{\rm{L}}}}}\, < \,{\bar{\mu }}_{{{{\rm{L}}}}}:= \frac{{\mu }_{{{{\rm{L}}}}}}{1\,+\,{(Q/{\theta }_{ql})}^{2}}\,+\,{\kappa }_{{{{\rm{L}}}}}$. If ${\beta }_{{{{\rm{H}}}}}\, > \,{\bar{\mu }}_{{{{\rm{H}}}}}$, there exists a steady state *E*_1_ = (H^*^, 0), and if ${\beta }_{L}\, > \,{\bar{\mu }}_{{{{\rm{L}}}}}$, there exists a steady state *E*_2_ = (0, L^*^), where ${H}^{* }={\theta }_{{{{\rm{H}}}}}\left(\frac{{\beta }_{{{{\rm{H}}}}}}{{\bar{\mu }}_{{{{\rm{H}}}}}}-1\right)$ and ${L}^{* }=\frac{{\theta }_{{{{\rm{L}}}}}}{{\rho }_{2}}\left(\frac{{\beta }_{{{{\rm{L}}}}}}{{\bar{\mu }}_{{{{\rm{L}}}}}}-1\right)$. The steady state *E*_1_ = (H^*^, 0) (or *E*_2_ = (0, L^*^)) is locally asymptotically stable if H^*^ > *ρ*_2_L^*^ (or H^*^ < *ρ*_1_L^*^).

When the fusion gene formation rate *η* is non-zero, the system (5) has three possible steady states:

1. There always exists a trivial steady state *E*_0_ = (0, 0), which is locally asymptotically stable if ${\beta }_{{{{\rm{H}}}}} \,< \,{\bar{\mu }}_{{{{\rm{H}}}}}+\eta$ and ${\beta }_{{{{\rm{L}}}}}\, < \,{\bar{\mu }}_{{{{\rm{L}}}}}$.
2. If ${\beta }_{L} \,> \,{\bar{\mu }}_{{{{\rm{L}}}}}$, there exists a leukemia-dorminant steady-state *E*_2_ = (0, L^*^). This state is locally asymptotically stable if ${\theta }_{{{{\rm{H}}}}}\left(\frac{{\beta }_{{{{\rm{H}}}}}}{{\bar{\mu }}_{{{{\rm{H}}}}}+\eta }-1\right)\, < \,{\theta }_{{{{\rm{L}}}}}\left(\frac{{\beta }_{{{{\rm{L}}}}}}{{\bar{\mu }}_{{{{\rm{L}}}}}}-1\right)$.
3. If ${\theta }_{{{{\rm{H}}}}}\left(\frac{{\beta }_{{{{\rm{H}}}}}}{{\bar{\mu }}_{{{{\rm{H}}}}}+\bar{\eta }}-1\right)\, > \,{\theta }_{{{{\rm{L}}}}}\left(\frac{{\beta }_{{{{\rm{L}}}}}}{{\bar{\mu }}_{{{{\rm{L}}}}}}-1\right)$, the HSPCs and LSPCs coexist, and there is a positive steady-state $\hat{E}=(\hat{H},\hat{L})$, where $\hat{H}$ and $\hat{L}$ satisfy
  $$\left\{\begin{array}{l}\frac{{\beta }_{{{{\rm{H}}}}}}{1\,+\,(\hat{H}\,+\,{\rho }_{1}\hat{L})/{\theta }_{{{{\rm{H}}}}}}=({\bar{\mu }}_{{{{\rm{H}}}}}\,+\,\eta ),\quad \\ \frac{{\beta }_{{{{\rm{L}}}}}}{1\,+\,(\hat{H}\,+\,{\rho }_{2}\hat{L})/{\theta }_{{{{\rm{L}}}}}}={\bar{\mu }}_{{{{\rm{L}}}}}-\eta (\hat{H}/\hat{L}).\quad \end{array}\right.$$
  Moreover, the state $\hat{E}$ is locally asymptotically stable.

Supplementary Fig. 3 shows the bifurcation diagram with respect to changes in *η* and *Q*. Supplementary Fig. 3a indicates that HSPC and LSPC coexist when *Q* and *η* are small values. Supplementary Fig. 3b, c illustrate the dependence LSPC ratio on *η* and *Q*, respectively.

From the bifurcation analysis, we need the fusion gene formation rate *η* larger than 10^−3^ during CML initiation and later decrease to a value below 10^−5^ after TKI treatment in order to describe the clinical observations from disease progression to treatment-free remission (TFR).

### Parameter estimation

To estimate the model parameters, we placed biologically plausible restrictions. Leukemia cells are expected to have a higher proliferation rate and a lower death rate compared to normal cells. Therefore, we set *β*_L_ > *β*_H_ and *μ*_L_ < *μ*_H_^46^. Additionally, the death rates in peripheral blood were assumed to be larger than those in the bone marrow, leading to the restrictions *μ*_PH_ > *μ*_H_, *μ*_PL_ > *μ*_L_. These assumptions resulted in the following restrictions on the model parameters:

$$\begin{array}{l}{\mu }_{{{{\rm{H}}}}}\, > \,{\mu }_{{{{\rm{L}}}}},\quad {\beta }_{{{{\rm{H}}}}}\, < \,{\beta }_{{{{\rm{L}}}}},\quad {\mu }_{{{{\rm{PH}}}}}\, > \,{\mu }_{{{{\rm{H}}}}},\\ {\kappa }_{{{{\rm{H}}}}}\, \sim \,{\mu }_{{{{\rm{H}}}}},\quad {\kappa }_{{{{\rm{L}}}}}\, \sim \,{\mu }_{{{{\rm{L}}}}},\quad {\mu }_{{{{\rm{PL}}}}}\, > \,{\mu }_{{{{\rm{L}}}}}.\end{array}$$

Here ~ means the same order of magnitude.

We referred to previous studies^46,71^ on hematopoietic stem cells and CML progression dynamics to identify the key parameters for stem cell proliferation, differentiation, and apoptosis rates. These studies provided experimental data and mathematical models that helped us estimate these parameters.

The cell kinetic rates for hematopoietic stem cell regeneration dynamics can be estimated based on a G0 cell cycle model using the technique of continuous labeling^71^. It was found that in mice, the differentiation rate ranges between about 0.01 and 0.02, the rate of cell re-entry from G0 back into the proliferative phase is between 0.02 and 0.05, and the rate of apoptosis from the proliferative phase is between 0.07 and 0.23 (all units are days^−1^).

In a previous study^46^, the CML dynamics were studied with a four-compartment model, and the kinetic parameters were compared with patients receiving imatinib treatment. The treatments lead to a biphasic exponential decline of leukemia cells. The first slope of 0.05 per day represents the turnover of differentiated leukemic cells, while the second slope of 0.008 per day represents the turnover rate of leukemic progenitors. For patients who relapsed after stopping imatinib treatment, the rapid upslope of 0.09 ± 0.05 per day corresponds to a doubling time of roughly 8 days^46^.

Based on these data, we assumed the following ranges of kinetic rates of hematopoietic stem cells:

$${\beta }_{{{{\rm{H}}}}},{\beta }_{{{{\rm{L}}}}} \sim 1{0}^{-1}{{{{\rm{day}}}}}^{-1},{\mu }_{{{{\rm{H}}}}},{\mu }_{{{{\rm{L}}}}} \sim 1{0}^{-3}{{{{\rm{day}}}}}^{-1},{\kappa }_{{{{\rm{H}}}}},{\kappa }_{{{{\rm{L}}}}} \sim 1{0}^{-3}{{{{\rm{day}}}}}^{-1}.$$

Next, we randomly sampled parameter values across a wide range of parameter space. We identified the parameter values that could produce the three phases of CML evolution dynamics. The acceptable dynamics include a chronic phase characterized by a slowly increased ratio of leukemia cells in the first few years following the onset of fusion gene formation, an accelerated phase with a rapid increase in the ratio, and finally, a crisis phase with a high level of leukemia cell ratio.

Parameter values used in the current study are listed in Supplementary Table 1.

### Numerical scheme

The numerical solution of the model equations was obtained using Euler’s method with a time step Δ*t* = 0.05. The number of leukemia cells produced from normal stem cells was determined by a Poisson random number Ψ([PSHC], *η*) at each time step. In simulations, we initialized the system with the following initial condition at *t* = −5 years:

$$[{{{\rm{HSPC}}}}]=9\times 1{0}^{6},[{{{\rm{LSPC}}}}]=0,[{{{\rm{PBHC}}}}]=14\times 1{0}^{6},[{{{\rm{PBLC}}}}]=0,Q=0.$$

The mutation rate, *η*_0_(*t*), was negligible when *t* < 0. As a result, the system approached a steady state without leukemia cells at *t* = 0, and the steady-state values are insensitive to the initial values. However, when *t* > 0, *η*_0_(*t*) increase rapidly to a high level, indicating the leukemia cells were introduced through the Poisson random number Ψ([PSHC], *η*). The time point *t* = 0 corresponds to when leukemia stem cells appeared.

### Virtual patients and overall survival

To validate the model, we generated virtual patients and defined their probability of mortality to fit the clinical overall survival curve.

We assumed that BCR-ABL1 generation in different patients has heterogeneous origins, leading to different values of the rate function *η*_0_(*t*) for each patient. This rate function was defined by uniform random numbers (*t*_1_, *τ*_1_, *t*_2_, *τ*_2_). Furthermore, the kinetic rates of leukemia cell proliferation (*β*_L_), apoptosis (*μ*_L_), and differentiation (*κ*_L_) were randomly chosen for each patient.

To compare the simulation results with clinical survival data, we assumed a mortality probability dependent on the PBLC ratio *R*_PBLC_. The formulation for this probability was given by:

6 $${P}_{{{{\rm{death}}}}}({R}_{{{{\rm{PBLC}}}}})=\frac{{P}_{0}}{1+{e}^{-({R}_{{{{\rm{PBLC}}}}}-{\mu }_{p})/{\sigma }_{p}}}.$$

Here, *R*_PBLC_ is the ratio of PBLC to the sum of PBLC and PBHC numbers, represented as

$${R}_{{{{\rm{PBLC}}}}}=\frac{[{{{\rm{PBLC}}}}]}{[{{{\rm{PBLC}}}}]+[{{{\rm{PBHC}}}}]}.$$

The term *P*_death_(*R*_PBLC_)Δ*t* represents the mortality probability of a patient with a PBLC ratio of *R*_PBLC_ in a time interval [*t*, *t* + Δ*t*].

To adjust the model parameters, we used the survival curve of untreated patients. We referred to an MD Anderson Cancer Center dataset that included 1569 patients with chronic myeloid leukemia (CML) since 1965. Of them, 140 patients received no treatment (diagnosed before 1975)^60^. All the patients were grouped based on the years of therapy, and the overall survival probability curves were provided for each subgroup. To identify the model parameters, we initially set *d*_1_ = *d*_2_ = *d*_3_ = 0 and used the least-squares method to fit the survival curve for the group of 140 patients that received no treatment. The cost function used for fitting was defined as:

$${{{\rm{cost}}}}=\frac{1}{N}\mathop{\sum }\limits_{i=1}^{N}{({P}_{i,{{{\rm{simulation}}}}}-{P}_{i,{{{\rm{clinical}}}}\,{{{\rm{data}}}}})}^{2},$$

where *P*_*i*_ represents the survival probability at the *i*^th^ year after diagnosis.

Next, we tuned the treatment effects (*d*_1_, *d*_2_, *d*_3_) to achieve an overall survival rate of about 80% at 10 years after diagnosis, as referred to clinical studies^60,62^. The dataset presented in Hehlmann (2016)^62^ included the survival rates of patients with CML in five consecutive randomized studies of the Germain CML Study since 1983, updated in 2016. The corresponding survival curve is shown in Fig. 3a.

### Thresholds of TME index and LSPC for CML relapse

We aimed to determine the TME index and LSPC number threshold to predict patient responses after treatment discontinuation. To achieve this, we assumed that the tumor microenvironment is in a quasi-steady state by setting the derivative $\frac{{{{\rm{d}}}}Q}{{{{\rm{d}}}}t}$ equal to zero. Using this assumption, we derived the following equation that relates TME index (*Q*) and LSPC ([LSPC]):

7 $${\kappa }_{{{{\rm{Q}}}}}\frac{{([{{{\rm{LSPC}}}}]/\theta )}^{n}}{1+{([{{{\rm{LSPC}}}}]/\theta )}^{n}}(1-Q)-{\kappa }_{{{{\rm{I}}}}}\frac{Q}{1+{(Q/{\theta }_{q})}^{s}}=0.$$

From (7) and given the parameters *κ*_*Q*_, *κ*_*I*_, *θ*, *θ*_*q*_, *s*, and *n*, we can solve *Q* as a function of [LSPC], which is shown by Supplementary Fig. 4. From Supplementary Fig. 4, there is a critical value (marked by the black dot) so that the TME index switches to a large value when the LSPC number is larger than the critical level.

Expressing [LSPC] as a function *Q* from equation (7) gives the following relation

8 $$[{{{\rm{LSPC}}}}]=\theta {\left(\frac{{\kappa }_{I}Q}{{\kappa }_{Q}(1-Q)(1+{(Q/{\theta }_{q})}^{s})-{\kappa }_{I}Q}\right)}^{\frac{1}{n}}.$$

The critical point is given by the local maximum [LSPC] in accordance with *Q*, which is determined by $\frac{{{{\rm{d}}}}[{{{\rm{LSPC}}}}]}{{{{\rm{d}}}}Q}=0$. A simple calculation implies that the critical value *Q* satisfies the equation

$${\left(\frac{{\kappa }_{I}Q}{{\kappa }_{Q}(1-Q)[1+{(Q/{\theta }_{q})}^{s}]-{\kappa }_{I}Q}\right)}^{\frac{1}{n}}\cdot \frac{{\kappa }_{Q}(1+(sQ-s+1){(Q/{\theta }_{q})}^{s})}{nQ({\kappa }_{Q}(Q-1){(Q/{\theta }_{q})}^{s}+({\kappa }_{I}+{\kappa }_{Q})Q-{\kappa }_{Q})}=0,$$

or

9 $$1+(sQ-s+1){(Q/{\theta }_{q})}^{s}=0.$$

The critical value *Q* depends only on the parameters *θ*_*q*_ and *s*. In particular, when *θ*_*q*_ = 0.11 and *s* = 2 in our simulations, we have the critical value *Q*_1_ = 0.127, which is shown by the black dashed line in Fig. 4c.

### Reporting summary

Further information on research design is available in the Nature Research Reporting Summary linked to this article.

### Supplementary information

Supplementary Information

Reporting Summary

## Data Availability

All data generated and analyzed during this study are included in this article. The datasets generated during the current study are available from the corresponding author upon reasonable request.

## Code availability

The MATLAB codes are available from the corresponding author upon reasonable request.
