# Supplementary material for: Computational modeling reveals key factors driving treatment-free remission in chronic myeloid leukemia patients
Source: NPJ Syst Biol Appl. 2024 Apr 27;10:45. doi: 10.1038/s41540-024-00370-4 (PMC11055880; doi:10.1038/s41540-024-00370-4)
Supplement: Supplementary file 1 — Supplementary Information [file 41540_2024_370_MOESM1_ESM.pdf]

Supplementary Information

Supplementary Table 1: Parameter values of the model

Parameter	Description	Value	Unit
β_{H}	Basic proliferation rate of HSC	0.112	day^{-1}
θ_{H}	Effective level for HSPC inhibition by HSPC and LSPC	1.8×10^6	cells/kg
ρ_1	Ratio factor	1	-
μ_{H}	Death rate of HSPC	0.007	day^{-1}
κ_{H}	Differentiation rate of HSPC	0.0058	day^{-1}
μ_{PH}	Death rate of PBLC	0.0088	day^{-1}
θ_{qh}	Effective TME level for promotion of HSPCs apoptosis	0.1	-
μ_0	Maximum Promotion rate of HSPC apoptosis by TME	0.5	-
α_{H}	Amplification rate of HSPCs	12	-
θ_{L}	Effective level for LSPC inhibition by HSPC and LSPC	0.567×10^6	cells/kg
ρ_2	Ratio factor	1	-
θ_{ql}	Effective TME for inhibition of LSPC death	0.25	-
μ_{PL}	Death rate of PBLC	0.006	day^{-1}
α_{L}	Amplification rate of LSPC	24	-
κ_{I}	Rate of restoring NME from TME	0.015	day^{-1}
θ	Effective level of LSPC in promoting NME to TME transition	2.0×10^6	day^{-1}
δ_{L}	Maximum production rate of LSPC from HSPC	0.0045	day^{-1}
θ_a	Effective TME level in the production rate of LSPC from HSPC	0.2	-
ε_0	Fraction of residual transformation rate of HSPC to LSPC	0.0315	day^{-1}
θ_q	Effective TME level in inhibition of TME depredation	0.11	-
m, n, s	Hill coefficient	2	-
β_L	Basic proliferation rate of LSPC	$[0.1557, 0.1903]$	day^{-1}
μ_L	Apoptosis rate of LSPC	$[0.0054, 0.0066]$	day^{-1}
κ_L	Differentiation rate of LSPC	$[0.0020, 0.0024]$	day^{-1}
κ_{Q}	Rate of transformation from NME to TME	$[0.0089, 0.0101]$	day^{-1}
t_1	Parameter relate to BCR-ABL1 occurrence $\eta_0(t)$	$[7, 9]$	year
τ_1	Parameter relate to BCR-ABL1 occurrence $\eta_0(t)$	$[4, 5]$	year
t_2	Parameter related to BCR-ABL1 occurrence $\eta_0(t)$	$[9, 13]$	year
τ_2	Parameter related to BCR-ABL1 occurrence $\eta_0(t)$	$[0.2, 1]$	year
P_0	Maximum value of death probability of a patient	1.0	day^{-1}
μ_P	Parameter related to the death probability of a patient	0.5	-
σ_P	Parameter related to the death probability of a patient	0.03	-
(d_1, d_2, d_3)	Drug effects	$(10, 10, 15)$	-

Supplementary Figures

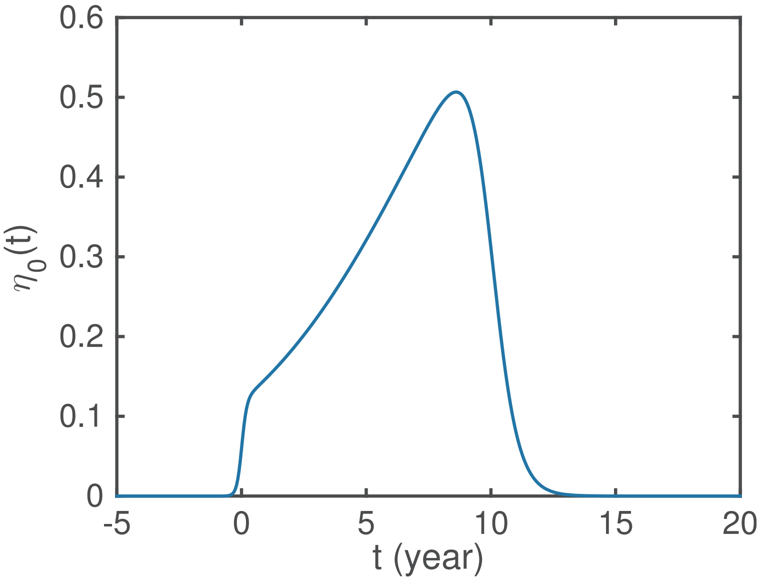

Supplementary Figure 1: The function $\eta_0(t)$. Here, $t_1 = 8, t_2 = 10, \tau_1 = 4, \tau_2 = 0.5$.

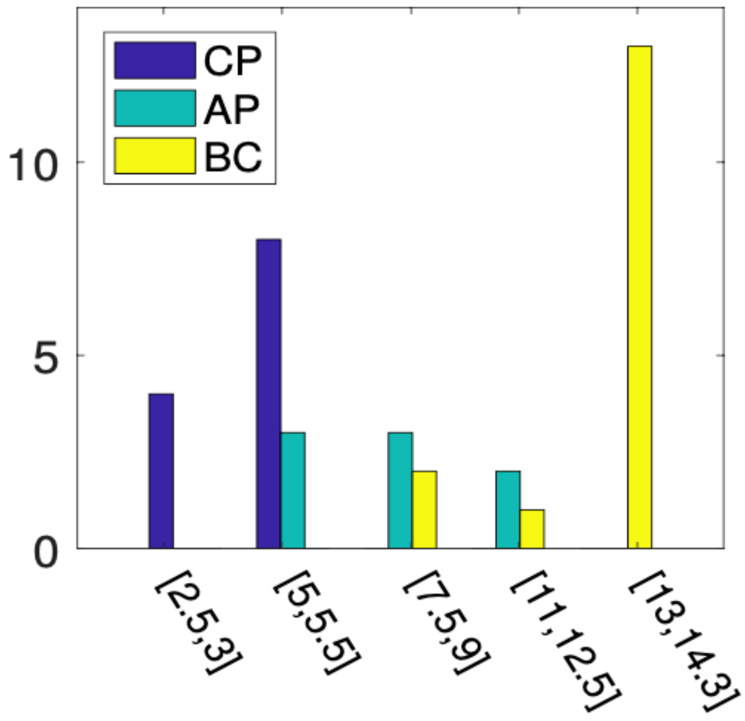

Supplementary Figure 2: Distribution of the disease ages of patients at CP, AP, and BC phases.

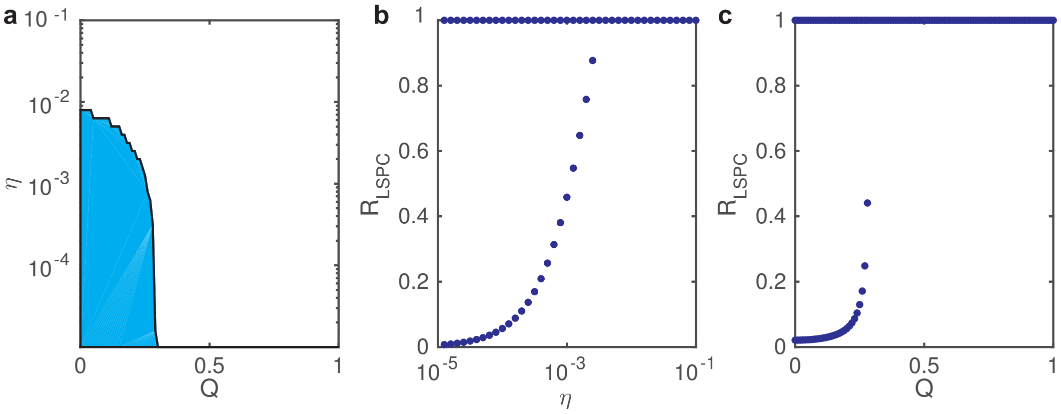

Figure Figure 3: Bifurcation diagram. **a**. Bifurcation with respect to parameters (Q, η). The cyan region indicates the coexistence of HSPCs and LSPCs, represented by the state $\hat{E} = (\hat{H}, \hat{L})$. **b**. LSPC ratio at the steady-state versus η, with $Q = 0.2$. **c**. LSPC ratio at the steady-state versus Q, with $\eta = 10^{-4}$. Here, the LSPC ratio is defined as $R_{\text{LSPC}} = \hat{L}/(\hat{H} + \hat{L})$ at the steady-state. Other parameters are the same as in Supplementary Table 1.

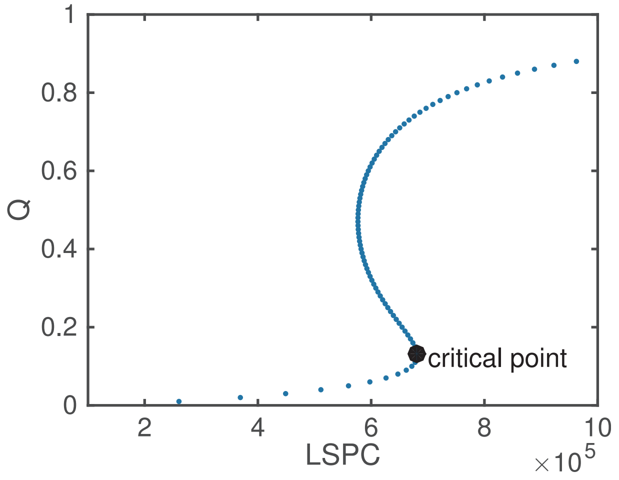

Supplementary Figure 4: Critical value for LSPC and TME index. Here, $\kappa_Q = 0.009$ and other parameters refer to Supplementary Table 1.

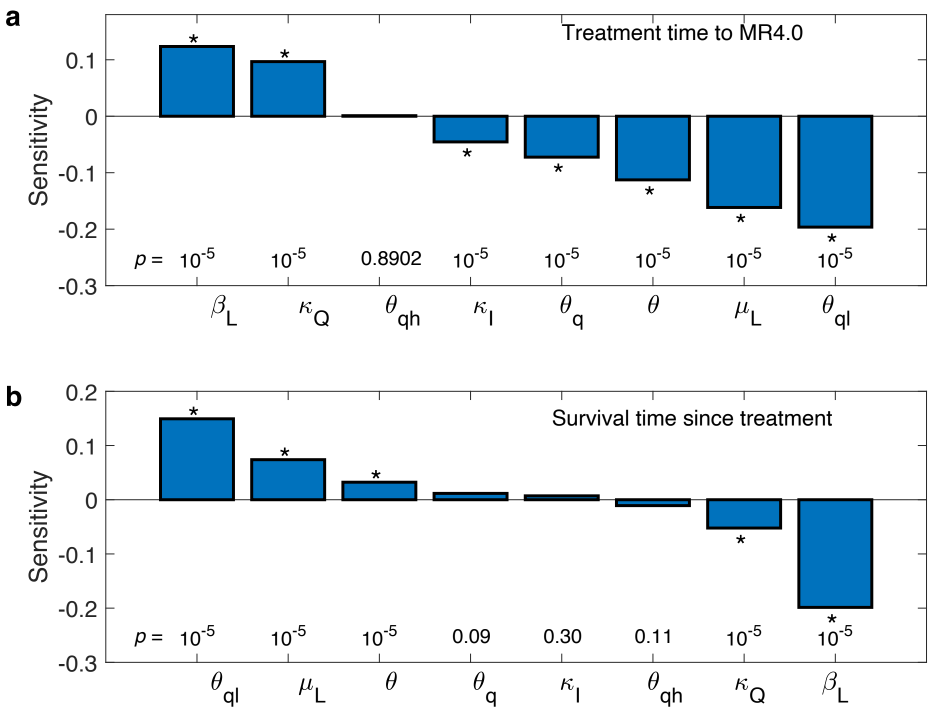

Supplementary Figure 5: Sensitivity analysis. a. Pearson's correlation coefficients of the treatment time to reach MR4.0 (for TFR patients) with various parameters. **b**. Pearson's correlation coefficients of survival time since TKI treatment (for patients who relapsed after stopping the treatment at MR4.0) with various parameters. Black stars indicate statistical significance with p-values less than 0.05 (t-test).

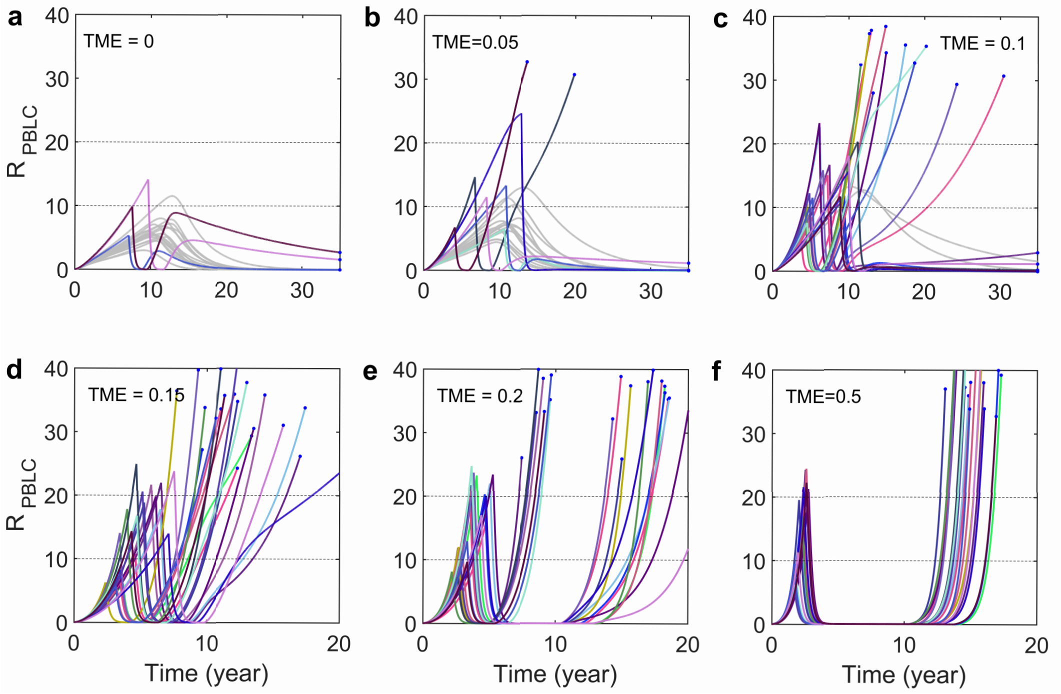

Supplementary Figure 6: Evolution of PBLC percentage after treatment discontinuation based on the model with constant TME. **a**-**f**: Evolution of PBLC percentage (R_{PBLC}) for 20 virtual patients with random initiation of TKI treatment when $5\% < R_{\mathrm{PBLC}} < 25\%$, and treatment discontinuation when PBLC percentage level reach $R_{\mathrm{PBLC}} = 0.01\%$, with constant TME $= 0,\ 0.05,\ 0.1,\ 0.15,\ 0.2$, and 0.5, respectively. In (**a**)-(**b**), some patients automatically develop into the chronic phase (CP) without treatment, which are shown by gray lines.

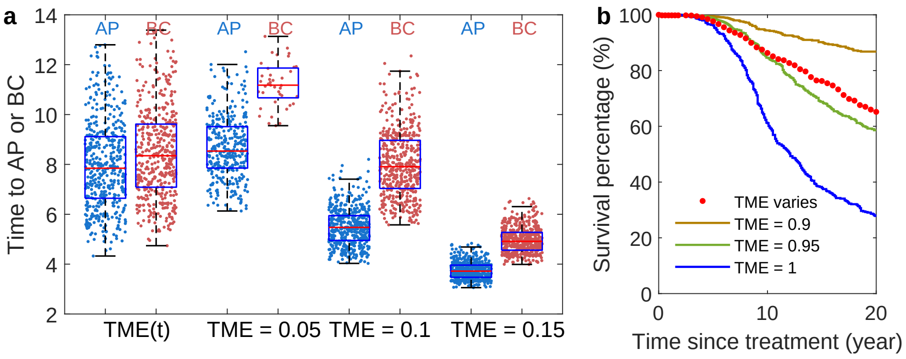

Supplementary Figure 7: Comparison of the time to AP and BC phases and survival curves. **a**. Comparison of the time to AP and BC phases for models with dynamically changing TME (TME(t)) or constant TME indices with TME $= 0.1, 0.15$, and 0.2, respectively. **b**. Survival curves of virtual patients after continuous TKI treatment. Parameters are the same as in Figure 3a. The dotted line shows the survival curve of virtual patients with dynamically changing TME based on the proposed stochastic differential equation model. The solid lines are survival curves obtained from constant TME $= 0.9, 0.95$, and 1.0, respectively.
